# The Diversity of N-Glycans of *Chlorella* Food Supplements Challenges Current Species Classification

**DOI:** 10.3390/foods13193182

**Published:** 2024-10-07

**Authors:** Réka Mócsai, Johannes Helm, Karin Polacsek, Johannes Stadlmann, Friedrich Altmann

**Affiliations:** Department of Chemistry, BOKU University, Muthgasse 18, 1180 Vienna, Austria; rekamocsai@gmail.com (R.M.); jhelm@gmx.net (J.H.); karin.polacsek@boku.ac.at (K.P.); j.stadlmann@boku.ac.at (J.S.)

**Keywords:** microalgae, *Chlorella*, N-glycan, glycoprotein, dietary supplements, food authenticity

## Abstract

N-glycans have recently emerged as highly varied elements of *Chlorella* strains and products. Four years and eighty samples later, the increasing N-glycan diversity calls for a re-examination in the light of concepts of species designations and product authenticity. N-glycans of commercial products were analyzed by matrix-assisted time-of-flight mass spectrometry (MALDI-TOF MS) supported by chromatography on porous graphitic carbon with mass spectrometric detection. Although 36% of 172 products were labeled *C. vulgaris*, only 9% presented what could be taken as a *C. vulgaris* type N-glycan pattern. Respectively, 5 and 20% of the products matched with *C. sorokiniana* strains SAG 211-8k and SAG 211-34, which, however, carry entirely different structures. Furthermore, 41% presented with one of four frequently occurring glyco-types while 26% of the samples showed unique or rare N-glycan patterns. These glycan signatures thus profoundly challenge the stated species designations. By no means do we want to question the presumed health benefits of the products or the sincerity of manufacturers. We rather aim to raise awareness of the fascinating but also concerning diversity of microalgal N-glycans and suggest it as a means for defining product identity and taxonomic classifications.

## 1. Introduction

The name *Chlorella* was given to a group of small green eukaryotic cells with hardly any obvious visual features for distinguishing genera and species [1,2,3,4,5]. Despite this, the NCBI taxonomy repository lists 42 genera within the *Chlorellaceae* family, and the genus *Chlorella* is divided into 25 species. The difficulty of unequivocal classification of microalgae is demonstrated by rather frequent re-classification events that sometimes even jump across class borders, as in the case of “*Chlorella fusca*”, which is now called *Scenedesmus vacuolatus* from the class of *Chlorophyceae* [6]. For the newcomer: contrary to first impressions, the *Chlorophyceae* are not the class that includes the *Chlorellaceae* family with its best-known member *Chlorella*.

These uncertainties did not prevent scientists from evolving a multitude of—in part already industry scale—applications of *Chlorella* and other microalgae [7,8,9,10,11]. In 2023, 619 PubMed entries contained the term *Chlorella* in a highly diverse array of contexts, reviewed e.g., by Abreu et al. [12]. Frequent topics include the production of biomass and lipids, sometimes with the scope of biodiesel and biohydrogen production [13,14,15]. These lower-value applications are often connected to objectives such as wastewater treatment, heavy metal, nitrogen or phosphorus depletion and—last but not least—carbon sequestration [15,16,17]. A remarkable niche application is the expression of antigenic protein for the vaccination of shrimps [18]. Higher priced products for human consumption comprise dyes such as astaxanthin or phycocyanin, unsaturated fatty acids (omega-3 fatty acids), vegan protein and vitamins [19,20,21]. An enormous growth potential is attributed to the food market sector of *Chlorella* and other edible microalgae [21,22]. A natural, vegan source of vitamin B12 certainly is of interest [23,24], whereby caution is advised as older determination methods could not distinguish between true vitamin B12 and its biologically inactive pseudo-vitamin that prevails in *Spirulina* [25]. Recent studies found rather varying contents of B12 in a panel of *Chlorella* samples [25]. This may well reflect the diverse nature of the microalgae constituting the products as demonstrated by their differing N-glycosylation. In any case, the cobalamin content appears to be the best substantiated of the many health claims for *Chlorella* dietary supplements. In contrast, a scientific basis for the likewise often acclaimed benefit of the high chlorophyll content of microalgae has not been brought to the attention of the authors of the present study.

Green algae, and thus also *Chlorella*, are eukaryotic organisms, which render many of their proteins with asparagine-linked oligosaccharides, so-called N-glycans [26,27,28]. Concepts about the physiological role of these glycans in land plants are only just emerging [29]. Quite certainly, however, all land plants contain the very same set of N-glycan structures for which a highly conserved handful of glycosyltransferases is responsible [27].

**Figure 1:**
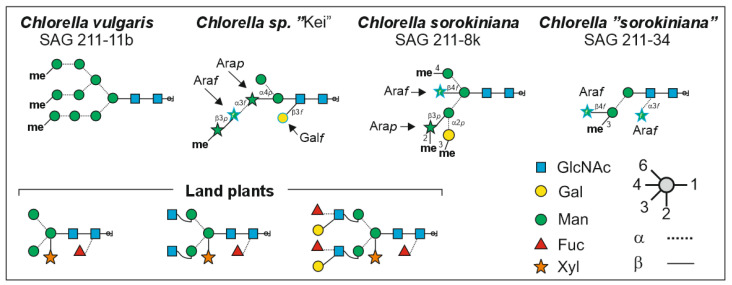
Structures of N-glycans from *Chlorella* (clade) strains known to date [31,32,34] in comparison with the major complex-type N-glycans that occur in all land plants [27,28]. Ara*f* and Ara*p* tand for arabinofuranose and arabinopyranose, hitherto not found in any other eukaryotic organism’s N-glycans.

Recently, we found that different culture collection lines as well as *Chlorella* nutraceuticals contained a range of different N-glycans and N-glycan patterns when viewed by mass spectrometry [30]. Very few products exhibited the pattern consistent with the *C. vulgaris*-type strain SAG 211-11b with methylated oligomannosidic N-glycans only. Several products displayed a *C. sorokiniana* [31] and others a “FACHB31“ pattern [32], each with very characteristic major glycans (Figure 1). Overall, the 80 products could be divided into at least 10 different glyco-types [30]. Notably, 22 of these products were labeled “*C. pyrenoidosa*”, which has been an invalid species designation for years already [6,33]. Despite this, PubMed contains 70 publications on *C. pyrenoidosa* only for the current year (as of 19 September 2024), many of which lack an explicit strain number.

The development of this field deserves interest and therefore we purchased and analyzed another 90 *Chlorella* products, including several re-purchases. We observed a few recurring glycan patterns alongside a remarkable number of novel patterns. We try to bring some order into the many glycan patterns as surfaced by matrix-assisted laser-desorption time-of-flight mass spectrometry (MALDI-TOF MS) together with a strategy for distinguishing isobaric N-glycans with the help of retention-time standardized liquid chromatography electrospray-ionization mass spectrometry on porous graphitic carbon (PGC-LC-MS). While this study casts doubts on the current practice of *Chlorella* strain designation, it also points at strategies for improved product characterization and quality control.

## 2. Materials and Methods

### 2.1. Chlorella Samples

Commercial *Chlorella* dietary supplements were ordered online or bought at local stores. A list of product names, principal distributors and lot numbers can be found in Table S1. Live cultures of culture collection strains were grown photoautotrophically in Bold’s basal medium (without antibiotics) as described [34].

### 2.2. N-Glycan Preparation and Analysis by MALDI-TOF MS and MS/MS

N-glycans were prepared by the differential application of cation exchange before and after digestion with peptide:N-glycosidase A (PNGase A; Agilent, Santa Clara, CA, USA) as described [30,34]. Free N-glycans were co-crystallized with 2,5-dihydroxybenzoic acid and analyzed with an Autoflex MALDI-TOF MS (Bruker, Bremen, Germany) in positive reflectron mode. Spectra were re-calibrated using oligomannosidic glycans as internal standards. Almost all signals qualified as representing N-glycans according to their composition of hexose, *N*-acetylhexosamine (HexNAc), pentose and methyl (which together might mask a deoxyhexose) residues as calculated by the spreadsheet “algae mass converter” (Table S2). A second criterion was the embedding in a cluster of similar compositions differing in, e.g., the number of methyl groups. Fragment spectra were obtained by laser-induced fragmentation (LIF) in “LIFT” mode and tentatively interpreted using the Excel spreadsheet tool (Table S3).

### 2.3. Analysis by Porous Graphitic Carbon Liquid Chromatography (PGC-LC-MS)

For analytical purposes, the glycans were reduced with NaBH_4_, desalted by passage over graphitized carbon cartridges (ThermoFisher Scientific, Vienna, Austria) and subjected to LC-MS with a PGC column (0.32 mm; 150 mm) connected to an amaZone Speed ion trap (Bruker) operating in data-dependent acquisition mode [30]. The PGC column was eluted with 65 mM ammonium formate at pH 3.0 and a gradient from 8 to 60% acetonitrile over 50 min at a flow rate of 6 μL/min at 30 °C. Retention times were normalized with the help of internal standards as described [35]. However, adapted to the current task, unlabeled porcine brain N-glycans and isotope-labeled diantennary glycan (^13^C_4_-A^4^A^4^F^6^), which was prepared from porcine fibrin exploiting the recently discovered de-*N*-acetylating potency of hydrazine hydrate [36,37], were used as internal standards. The respective measured retention times were converted to virtual minutes (v-min).

### 2.4. rDNA Barcoding

DNA extraction and PCR using primers devised to amplify the (18S)-ITS1-5.8S-ITS2-(23S) region was carried out as described in detail recently [30].

## 3. Results

### 3.1. A Roadmap through the Glycan Maze

Over the last four years, our collection of commercial *Chlorella* samples has been extended to now comprise 172 different samples, each with different brand names in vividly colored, attractive packaging. Most samples came from resellers who lacked the inclination to reveal the actual producer of a given lot. Thus, unfortunately, the sources of the products remain obscure. Products with very similar patterns may or may not stem from the same production site. On the other hand, it may happen—deliberately or not—that the production strain in a particular site changes over time. This may be the underlying reason for the observation that different lots from the same vendor exhibited different glycan patterns (13 out of 19 re-ordered samples gave a clearly different pattern; to avoid embarrassment, these data are not explicitly revealed herein). The same situation could, however, result from a change of supplier. A further source of confusion could be mixed production strains or blending of previously “pure” products. Despite these regrettable limitations of the current study, the matter deserves attention particularly in the light of food identity and authenticity.

Table S1 lists the products, their sources, lot numbers and the stated *Chlorella* species. The following sections try to structure the maze of variations and peculiarities. At first, we will deal with glycan patterns matching those of established culture collection strains. The remaining 76% of samples and their patterns will be split into four larger group, several smaller groups and finally into those with apparently unique patterns. This grouping is essentially based on mass spectrometry of N-glycans with some assistance from (18S)-ITS1-5.8S-ITS2-(23S) rRNA barcoding. In the following, relevant characteristics of the glycan pattern groups will be presented.

### 3.2. Vendors’ Designations of Chlorella Products

**Figure 2:**
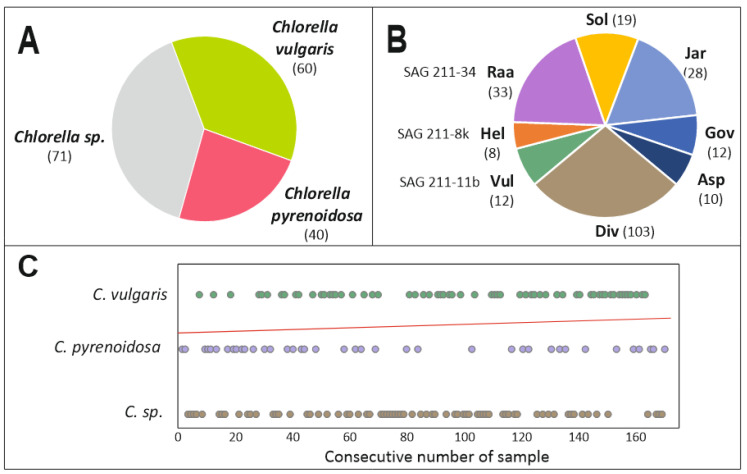
Product designations of commercial dietary *Chlorella* products. Panel (**A**) illustrates the distribution of product designations. Panel (**B**) depicts the frequencies of the glyco-patterns found in the study group of 172 *Chlorella* products. The arbitrary three letter abbreviations were introduced recently [30] and are further explained in the following sections and in Table 1. “Div” comprises all unique or rare patterns possibly including a few “misdiagnosed” ones. “Vul” bunches together spectra that at least remotely resemble the previously described *C. vulgaris* glycosylation pattern. Panel (**C**) shows the distribution of the product designations by sample number, which roughly reflects the purchase timeline over the last eight years. The red line indicates the trend in the frequency of the two species designations.

The food market in Europe is regulated. The *Chlorella* species that can be freely marketed as they do not fall under Novel Food Regulation 2283/2015 of the European Union are *C. vulgaris, C. pyrenoidosa* and *C. luteoviridis* (now renamed as *Jaagichlorella luteoviridis*) (https://www.algae-novel-food.com/output/algae-novel-food/download.pdf, accessed 6 September 2024). Among our collection of 172 samples, almost half of the products did not provide a species name, the rest being split up into *C. vulgaris* and *C. pyrenoidosa* (Figure 2, Table S1).

As our internal specimen numbers were assigned chronologically over a period of eight years, the use of species terms over time can be scheduled (Figure 2). A slight decrease in the popularity of the term *C. pyrenoidosa* can be sensed, which may be attributed to ever more producers or retailers realizing that this species name was officially abandoned by taxonomists and type strain collections [6]. One product was designated *C. sorokiniana*. This product came from the United States, where other regulations apply than in Europe.

### 3.3. Structuring the Pandemonium of MALDI-TOF MS N-Glycan Patterns

**Figure 3:**
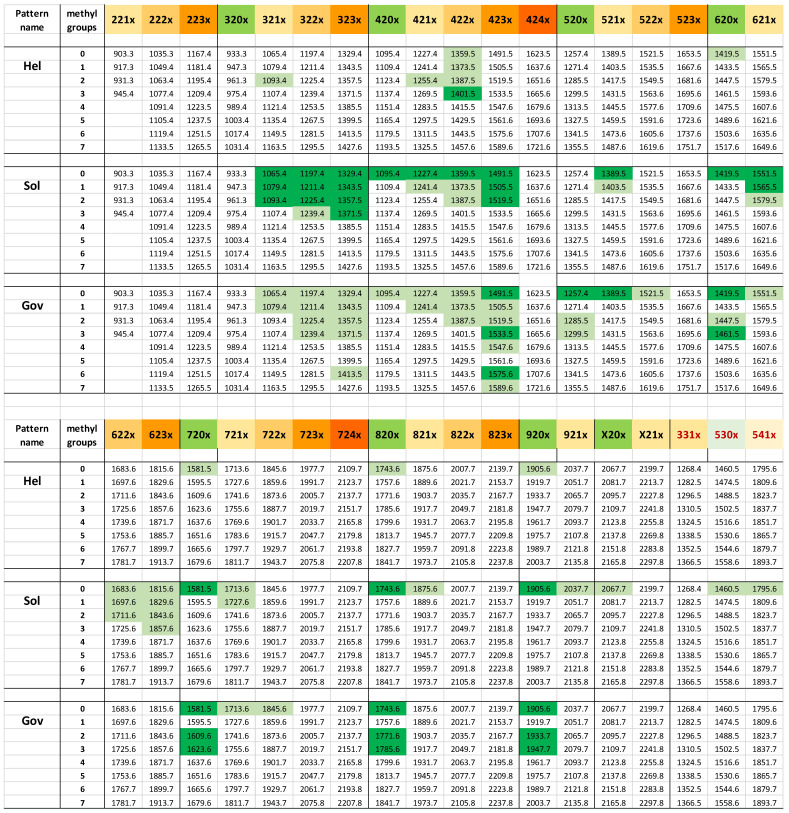
Microalgal mass map with three arbitrarily selected examples. The colored upper rows give the number of hexoses, HexNAcs and pentose residues in a structure. The rows below list the *m*/*z* values of [M + Na]^+^ ions with increasing numbers of methyl groups. Light and dark green cell colors represent smaller (approximately 5 to 30% of maximal peak height) and larger peaks. For a more detailed depiction, please refer to Table S4.

In this study, we focus on the N-glycan spectra derived from various products. Due to the extraction and detection procedures, these spectra display neutral N-glycans only. However, no acidic or zwitterionic N-glycans have been found in algae so far. The combination of varying numbers of hexoses, pentoses, methyl groups and even *N*-HexNAcs leads to a vast variety of mass values. Note, that this study does not identify deoxyhexoses, which are isobaric to the combination of a pentose and a methyl group, and which have so far not been found in *Chlorella* clade algae [30]. From the 132 counted mass values, 36 represent oligomannosidic glycans with and without methyl groups, 91 comprise pentose-containing N-glycans and 18 even contain three to four HexNAcs. The exact masses and examples of how samples could be depicted in a QR code-like manner are shown in Table S4 an d in Figure 3. Note that in the following text and in the main text figures, often only nominal masses are given, as the addition of 1, 2 or more digits behind the comma would be pointless. Compositions can be derived from masses by the spreadsheet “algae mass converter” (Table S2).

**Figure 4:**
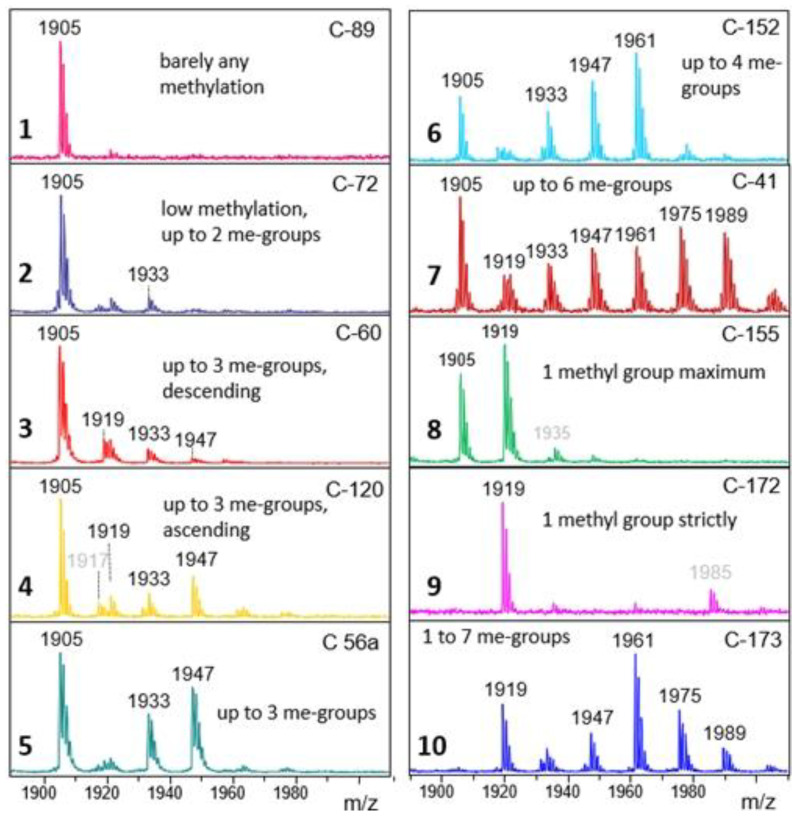
Ten characteristic patterns of O-methylation of the oligomannosidic N-glycan Man9 (Man_9_GlcNAc_2_). The numbers in the upper right corners refer to the respective *Chlorella* product (Table S1). Peaks are annotated with the nominal *m*/*z* values of the [M + Na]^+^ ions. Note that in this and all following spectra large peaks can be followed by a [M + K]^+^ satellite. Peaks annotated with gray color are unrelated structures.

**Figure 5:**
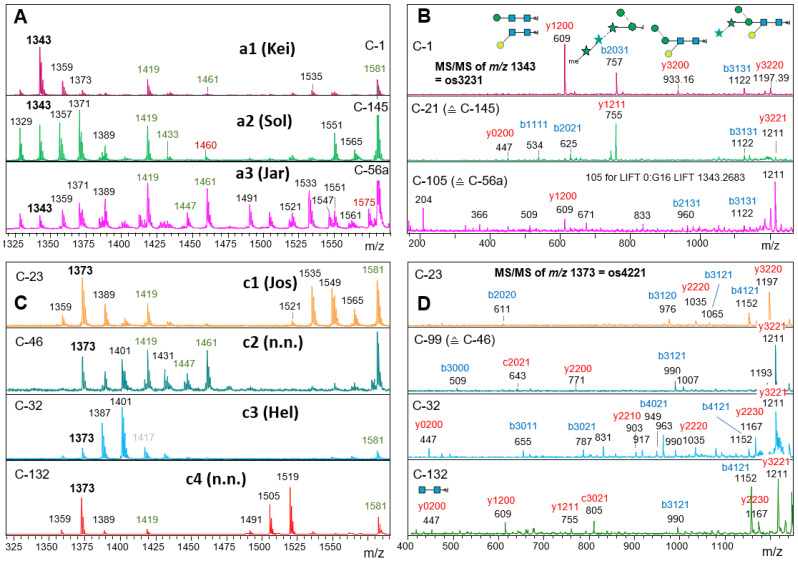
Seven distinct patterns of the medium mass range of microalgal N-glycans. MS1 spectra are shown on the left, LIF-MS/MS spectra of *m*/*z* = 1343.4 (panels (**A**,**B**)) and 1373.4 (panels (**C**,**D**)) on the right. In **A** and **C**, oligomannosidic glycans are annotated in green, K-adducts in grey and the low abundance indicator mass for “Sol” in red. Fragments are tentatively assigned as y (in red) and b (and c; in blue) ions with the numbers of hexose, HexNAc, pentose and methyl constituents. Cartoons are added where possible. The MS/MS spectra indicate different structures for all chosen precursor masses.

**Figure 6:**
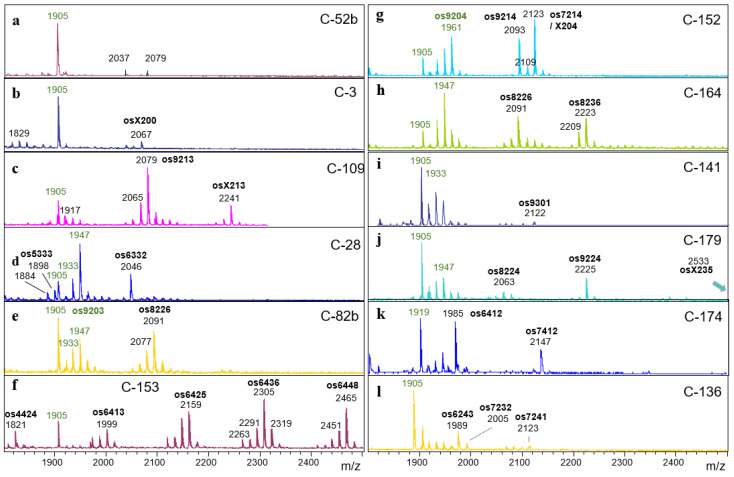
Twelve variant spectra of the high-mass region of *Chlorella* N-glycans. Oligosaccharides (os) are characterized by the numbers of hexose, HexNAc, pentose and methyl constituents, whereby X stands for 10. Oligomannosidic glycans are annotated in green,.

A depiction of full range spectra would necessarily conceal details. Therefore, just a few sections of particular diversity are shown. One shall be the Man9 region showing ten characteristic methylation patterns (Figure 4). A second example shall be the region *m*/*z* = 1300–1600 (Figure 5). As a third and last example, the high-mass region was chosen, which—beyond being impressively diverse—confirms the ability of some microalgae to generate glycans with up to four HexNAc residues (Figure 6).

A fourth and highly helpful criterion is the occurrence of a dominant “lead” mass (primarily *m*/*z* 1049, 1343 and 1401) that can be used to cluster product spectra. By a happy coincidence, the *m*/*z* values 1049 and 1401 represent the outstanding glycans of products for which culture collection strains are already known [31,32].

### 3.4. Commercial Samples with a C. vulgaris Pattern

With more than one third of all products being designated as *C. vulgaris*, one could expect an equally large number of samples to present an N-glycan pattern similar to that of the strains SAG211-11b, SAG 211-8l, UTEX235 [34] plus now SAG 211-80 and SAG 211-8m (Figure S1). The hallmark of these *C. vulgaris* strains is the exclusive presence of oligomannosidic N-glycans of which the larger part is equipped with up to six O-methyl groups [34]. This “type V” pattern of Man9 glycans was found in 14% of all samples. However, the majority of these products exhibited considerable amounts of additional peaks containing pentoses, possibly also deoxyhexoses and again methyl groups. Only 12 products could be accepted as having a rather “vulgaris-like” appearance. For one sample (C-126) DNA barcoding was performed and it corroborated the classification as *C. vulgaris* (Supplementary Sequences). However, almost all of these samples contained additional smaller peaks representing “pentose-glycans” (Figure S2). We cannot define whether these extra peaks are a previously overlooked feature of *C. vulgaris* or result from minor contamination of the cultures with other algae strains. At any rate, these extra peaks were highly varied. Another notable detail is that only two out of these 12 products carried the label *C. vulgaris*. Viewed from a different angle, 60 samples are labeled *C. vulgaris* but present a clearly different N-glycan pattern.

### 3.5. Samples with C. sorokiniana Patterns

Three *C. sorokiniana* strains (the type strain SAG 211-8 k and also SAG 211-32 and UTEX 1230) carried one very dominant glycan with structural elements not seen before [31]. Two other “*C. sorokiniana*” strains (SAG-211-31, SAG211-34 and also FACHB-31) surprised us with what almost resembled a number twister of the major peak´s mass (Figure S1). Instead of *m*/*z* = 1401, the dominant nominal mass here was 1049. At first sight, the small glycan could be a precursor or a degradation product of the larger one, but the structures of both N-glycans do not present any resemblance [32]. One can infer the number of specialized and unusual glycosyltransferases required to generate each of these glycans. With six and four such transferases needed to be bred by evolution just for these “*C. sorokiniana*” variants, we can no longer assume that these two glyco-types fit under the roof of the same species name. As, for the moment, we have to stick to these names, we will refer to these glyco-variants as “Hel” (*C. sorokiniana* with MALDI *m*/*z* = 1401) and “Raa” (*C. sorokiniana* and FACHB-31 with MALDI *m*/*z* = 1049) patterns, as in the previous publication [30]. Of particular note is the fact that while only one product was labeled *C. sorokiniana*, eight others had a “Hel” pattern and no less than 34 other samples had a “Raa” glycosylation (Figure 2, Table 1). Ironically, the one product labeled *C. sorokiniana* did not fit into either group. A further distinction of the “Raa” (*m*/*z* = 1049) group can be made based upon the methylation of Man9 (Table 1). Whatever the reason for these differences might be, LIF-MS/MS of the *m*/*z* = 1049 peak in all of these samples indicated identical glycan structures (Figure S3). Caution must, however, be exerted as the solitary sample C-5 gave a rather similar MS/MS spectrum despite a totally different structure [32]. LC-MS of selected samples advocated the notion of identical glycan structures (see Section 3.8 and Figure S4).

### 3.6. Frequently Found “Orphan” Glyco-Types

The term “orphan” encompasses glyco-types for which no culture collection strain has so far been identified. In the bewildering diversity of the often very crowded MALDI-TOF MS spectra of *Chlorella* N-glycans, three groups of densely nested signals were found in a large number of samples. These groups started with the masses 1197, 1329 and 1491 (Figure 5). These figures are the simplified integer *m*/*z* values of the [M + Na]^+^ ions observed by MALDI-TOF MS of released and purified N-glycans. A daunting complexity results from the addition of one or more methyl groups (14 mass units each). In some patterns, the oligomannosidic (OM) N-glycans were likewise methylated and contributed to signal plurality (Figure 4). As if not complex enough, potassium ions cause 16 mass unit increments—usually, but not always, with peaks much smaller than the main [M + Na]^+^ peak.

To get firm ground under our feet, we first established the possible composition of the mentioned masses. Our initial work had shown the hexoses mannose and galactose and the pentoses arabinose and xylose and, of course, HexNAc as the building blocks of the “club 1329” members [30]. A glycan mass can then be annotated in terms of the number of hexoses, HexNAc and pentoses and the number of methyl groups. A MALDI reading can be easily converted to a composition using the “algae mass converter” (Table S2). The [M + Na]^+^ value 1329 serves as an example for a glycan with three hexoses, two HexNAcs (i.e., GlcNAc) and three pentoses, and is annotated as os3230. *m*/*z* = 1343 then translates into os3231 and so on. The many combinations—realized or not by nature—can be listed in a spreadsheet as shown in Figure 3 and Table S4.

At least 74 samples adhered to the “1197, 1329 and 1491 plus methyl groups” schemes shown under the notations a2 or a3 in Figure 5. The samples differed in the occurrence and type of OM methylation and masses beyond *m*/*z* 2000. The distinction of samples with no or very little OM methylation (Man9 type I and II in Figure 4) could have been seen as futile were it not accompanied by the occurrence of *m*/*z* = 1460 (os5300) and 1795 (os5410) in the “Sol” group. Os5300 was shown to be the Man5Gn that forms the pivotal transition point from oligomannosidic to complex-type N-glycans in land plants and animals [30,37]. LIF-MS/MS of the *m*/*z* = 1343 peaks of samples exhibiting the a1 to a3 patterns substantiated the difference between patterns a2 (“Sol”) and a3 (“Jar”), as well as pattern a1, which is only found in the rare glyco-group “Kei” [30,31]. The a3 glyco-pattern was also seen in the more complex glyco-groups “Gov” and “Asp”, which unfortunately defied LIF-MS/MS. Their close relation to “Jar” was, however, substantiated by LC-ESI-MS (see Section 3.8 below). 

The small “Kei” group with its predominant *m*/*z* = 1343 shall be added here mainly because its major glycan structure has been elucidated [31].

At this stage, we had categorized 76% of the 172 products as either “Hel” (*m*/*z* = 1401), “Raa” (*m*/*z* = 1049), “Kei” (*m*/*z* = 1343) or the more complex glyco-groups “Sol” or “Jar-Gov-Asp” (Figure 1). The rest, unfortunately, involve a rather intricate variety of glycan-patterns.

### 3.7. Patterns Defying Classification

As much as 24% of the products could not—or at least not at first and second sight—be associated with any of the glyco-groups described above. Their features are listed in Table 1, whereby the primary sorting criterion was the occurrence of a dominant MALDI-TOF MS peak. Eleven different *m*/*z* values are seen here. A certain mass can derive from different structures in other samples, as revealed by LIF MS/MS. The example of *m*/*z* = 1343 has been discussed above (Figure 5). *m*/*z* = 1049 in C-5 reminds us of the “Raa” glyco-group, but stands for a different structure [32]. *m*/*z* = 1269 is remarkable as in C-46 this glycan was shown to contain fucose [30]. The new product C-152 exhibited the same MS/MS profile, whereas it differed in C-82 (Figure S5).

Next, samples were sorted according to their type of Man9 profile (Figure 4). Another sorting criterion was the pattern of high-mass N-glycans, which displayed a startling diversity (Figure 6). Another notable criterion was the presence of glycans with three or four HexNAc residues, which come with various compositions and apparently do not form a coherent group of structures. Finally, samples may exhibit characteristic peaks of moderate intensity. These are mentioned in a necessarily arbitrary and incomplete manner in Table 1. All spectra are shown in Figure S6.

### 3.8. Towards a Key Figure Characterization with PGC-LC-MS

**Figure 7:**
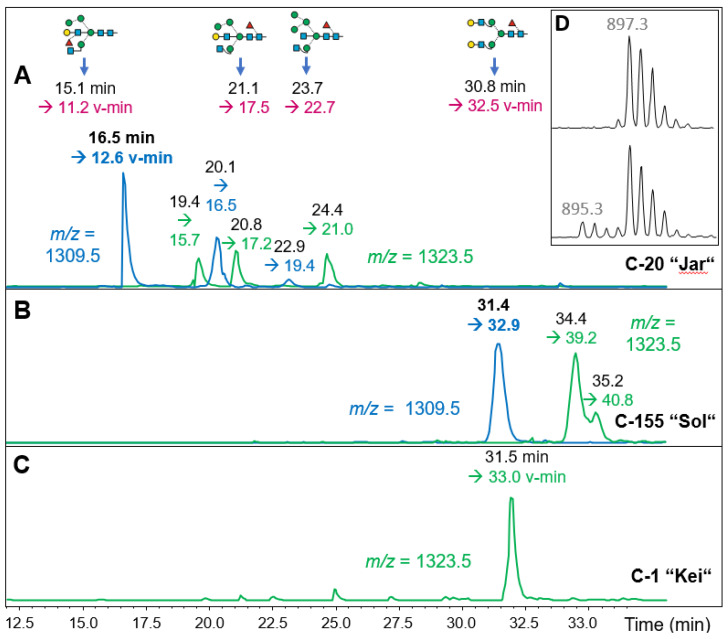
PGC-LC-ESI-MS with conversion to standardized retention times (v-min). Panel (**A**) shows the extracted ion chromatograms for os3230 and 3231 for a “Jar”-type product (C-20) together with the elution positions of internal standards obtained from porcine brain. Panel (**B**) depicts the elution of os3230 and 3231 in a product with a “Sol”-type pattern. These elution characteristics were also found for several other samples (C-15, C25, C-43 for “Jar” and C-21, C-89 and C-125 for “Sol”). Chromatogram (**C**) shows that the major glycan of the “Kei” sample C-1 does not co-elute with the isobaric glycans of “Jar” and “Sol”. Arrows at the top indicate the elution times of *m*/*z* = 895.3 brain glycans [35] and ^13^C_4_-labeled A^4^A^4^F^6^ [36,37], which were used to calculate v-mins (colored) from the initially recorded values (black). Insert (**D**) shows the spectrum of the isotope-labeled standard A^4^A^4^F^6^ alone or as part of a sample. Experimental retention times (v-mins) are written in black, standardized retention times in colored font.

Samples from the same or closely related *Chlorella* strains may—for various reasons—yield MALDI-TOF MS N-glycan patterns with varying peak heights. This “intra-sample” variance reduces the reliability of assignment. LIF-MS/MS spectra, informative as they are, are clumsy and waste space and, worst, often come as hybrid spectra. Instead, we propose retention-time normalized chromatography on porous graphitic carbon (PGC) as an approach to arrive at numbers that consider structure in addition to molecular mass. To make such retention times useful beyond the narrow scope of this study, they will be expressed as “virtual minutes” (v-min) with the help of isotope-labeled internal standards [35,36]. MALDI- or ESI-based MS/MS spectra can serve to corroborate or falsify an identity insinuated by equal (virtual) retention times. Having served this purpose, these spectra are no longer relevant as descriptors.We subjected selected samples containing peaks with *m*/*z* = 1329 to this PGC-LC-MS regime. One striking result was that the glyco-types “Jar”, “Gov”, “Asp” and even “Pit” exhibited identical retention times, whereas “Sol” samples clearly behaved differently (Figure 7). Thus, the subtle differences seen in the MALDI-TOF MS spectra, in particular the occurrence of *m*/*z* = 1460, were based on fundamental structural differences of the complex-type N-glycans of these samples, even though they consist of the same monosaccharide components as recently shown [30]. The methylated glycans of *m*/*z* = 1343 eluted after the parent peak, but did not co-elute with that mass from sample C-1 (Figure 7). So, obviously, neither the “Sol” nor the “Jar” and “Gov” glycotypes share the structure found for the C-1 (and C-54, 115, 151 and 108) major glycans.

Surprising in the contrary sense was the observation that *m*/*z* = 1329 of C-89, C-125 and C-155 co-eluted with typical “Sol” samples despite patterns that at first sight lacked an obvious resemblance with the “Sol” glyco-pattern. For consistent results, column temperature needs to be controlled carefully, as the adsorption isotherms of the internal standard and the microalga N-glycan apparently deviated to some extent.

As the MALDI-MS/MS patterns of C-5 and the “Raa” products´ *m*/*z* = 1049 differed only by peak ratios, and as the bona fide “Raa” products exhibited large differences in the larger glycans (Table 1), examples of each glyco-subtype were subjected to PGC-LC-MS. All *m*/*z* = 1029 (ESI equivalent to 1049 in MALDI spectra) peaks eluted at 17.3 v-min (Figure S3). Another example for the definition power of PGC-LC-MS was found in sample C-108. The MALDI peak of *m*/*z* = 1401 in C-108 and in “Hel” samples co-eluted on PGC (Figure S7). It may be noted that this glycan eluted extremely late, i.e., about 12 min after the glycan A^3^A^3^F^6^, which is the most retained member of the previously used standard series [35,37], indicating the identity of this structure. The glycan resembling the major “Kei” structure clearly contained one N-glycan, which co-eluted with the C-1 compound and also gave the same MS/MS spectrum (Figure S7). C-108 contained an additional two peaks with differing spectra (Figure S7), but this observation could be explained.

### 3.9. Contributions and Limits of DNA Barcoding

The primers used in the recent study fitted to the highly conserved ends of the 18S rRNA and 23S rRNA with optimal homology for *Chlorella*-clade algae [30]. In eleven cases (C- 6, 17, 21, 24, 28, 32, 35, 45, 59 and recently 126), these primers amplified a PCR product and, again in many cases, this PCR band gave a clear sequence without further subcloning (see “supplemental sequences” in [30]). Two products (C-36 and C-46) required sub-cloning. In these cases, 15 colonies (out of around 200) were randomly selected for sequencing. For many more products, no PCR product was obtained, which might reflect degradation of the DNA or too low a homology of the chosen primers with the sample, which in turn would point to a low kinship of the respective alga with the *Chlorellaceae*.

**Figure 8:**
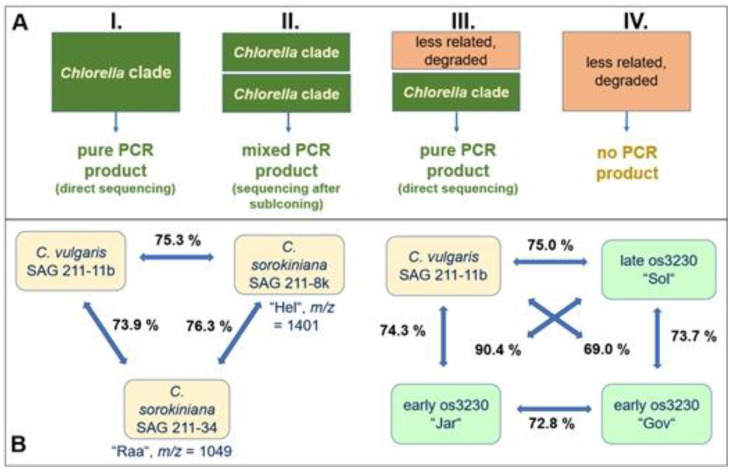
DNA barcoding of *Chlorella* products. (**A**): Scenarios of DNA barcoding with primers designed for *Chlorella*-clade microalgae. One rectangle symbolizes a product derived from one microalga strain. Peach-colored background indicates a strain that gave no PCR product. (**B**): Sequence identities of the ITS1-5.8S-ITS2 region (Supporting Information; Sequences) of selected samples. The comparison on the left involves strains with known, highly deviating glycan structures. The right block compares strains with partially identical features, i.e., oligomannose methylation in *C. vulgaris* and “Gov” on the one hand and the structure of os3230 (*m*/*z* = 1329) in “Jar” and “Gov” on the other. The attributes “early” and “late” refer to the elution times of the respective glycans. A complete identity matrix is presented in Figure S7.

In the case of C-6, C-32 and C-126, where a corresponding live strain was available, the glycan patterns of products and live strains matched almost perfectly. This is a highly important result as it demonstrates a strong link between genome type and N-glycan pattern in products that are derived from one alga strain (scenario A in Figure 8). The other products that gave one barcoding sequence might likewise belong to this group. They may, however, also fall into category C, where two or more algae species constitute the product, of which only one is amplified. This concept could explain why many products of the large-similarity groups (“Raa”, “Sol”, “Jar”, “Gov” and “Vul”) presented with variability in e.g., the type of methylation of oligomannosidic structures or the occurrence of high-mass glycans.

A brief look at the homologies of the (18S)-ITS1-5.8S-ITS2-(23S) barcodes of strains as well as products supports this notion. The sequence identities between *C. vulgaris* and two different *C. sorokiniana* strains (generating the “Hel” and the “Raa” patterns) are in the range of 75% (Figure 8). Together with their totally different N-glycan structures (Figure 1), the *C. sorokiniana* strains should probably be considered as two different species. The barcode dissimilarities between *C. vulgaris* and three frequent product types likewise indicates different species. Interestingly, there is no higher similarity between *C. vulgaris* and “Gov” on the one hand, as insinuated by oligomannose methylation, and between “Jar” and “Gov” on the other hand, as suggested by the identical structures of their “pentose-type” glycans (Figure 7). In contrast, “Sol” and “Gov” display a very high sequence identity despite different N-glycan structures (Figure 8).

With a sequence identity of 96.9%, the products C-1 (“Kei”) and C-23 are close to the point where they could be considered the same species [2]. Yet the differing N-glycans clearly speak another language (Figure 5). Figure S7 poses more such puzzles that await the findings for the corresponding alga strains for explanation.

## 4. Discussion

The analysis of the N-glycans of 172 commercial *Chlorella* products surprised us with a still expanding, seemingly unlimited variety of N-glycan patterns. At least 72% of the products fell into a few groups with obvious similarities in their MALDI-TOF MS patterns. In some cases, MALDI-TOF MS/MS and PGC-LC-MS could corroborate the identities, while in other cases differences were revealed as, e.g., between the “Sol” group and the—at first sight—rather similar supergroup comprising the “Jar”, “Gov” and “Asp” patterns. Some glyco-types may be further subdivided (e.g., the “Raa” group based on the degree of methylation of OM glycans), while others may be lumped together, such as the “Jar”, “Gov” and “Asp” groups, as they obviously share a common set of medium-sized pentose-containing glycans.

A frequent occurrence of a particular glyco-type may result from a particular strain being cultivated at different sites and times or it could be too many vendors obtaining raw material from just a few sources. Unfortunately, retailers are rarely inclined to reveal the source of their products. How far such discretion is appropriate in times of increasing demand for supply chain transparency is debatable. This lack of information about the products´ history leaves questions when we look at patterns in which different elements appear to be combined, such as, for example, the “Raa”-typical *m*/*z* = 1049 glycan occurring with a background of various types of methylation of oligomannosidic glycans.

Our recent DNA barcoding results revealed that glyco-type-based classification by and large matches genetic traits [30]. The big difference between DNA barcoding and N-glycan profiling lies in the innate ability of glycan profiling to provide a steadfast holistic view of a product´s content of eukaryotic organisms. The presence of a specific glycan structure or glycan pattern provides a clear-cut and unambiguous footprint of the production strain(s), whereas sequence differences in non-functional DNA are a somewhat nebulous matter. We propose that base exchanges in a region not subject to selection pressures are a less solid fundament for classifications than the formation of enzymes of clearly different functionality. Glycan-based characterization, by contrast, is independent of the quality of a product´s DNA or the primers used. It offers a more reliable portrayal of the eukaryotic components of a sample, even in the case of mixtures. N-glycan patterns or structures are newcomers in the toolbox of microalgae classification. Highly sophisticated tools for barcode assessments [5] and profound considerations of the proper application of DNA barcoding data already exist [2]. Therefore, hesitance towards implementation of yet another aspect is understandable, particularly given the considerable effort linked to it. We nevertheless believe that at least for strains of commercial or intense technical use, stakeholders should not shy away from incorporating this new tool.

For five glyco-types, the major N-glycans have been structurally elucidated (Figure 1) [31,32,34]. These glycans exhibited a number of features that had not yet been described, either in plants or in animal N-glycans. Each of these features necessitates a specific enzyme for its biosynthesis. Glyco-types that differ in several features thus require the emergence of several distinct glycosyltransferases. For example, the *m*/*z* = 1401 glycan in “Hel” samples requires three unusual glycosyl- and three specialized methyltransferases. Even the small *m*/*z* = 1049 “Raa” glycan could not be formed without three rather unique transferases. The spectra shown in Figure 4, Figure 5 and Figure 6 witness the existence of many more unusual transferases. This ingenuity is not restricted to the *Chlorella* clade as demonstrated by ongoing research on *Scenedesmus* N-glycans (R. Mocsai, manuscript in preparation).

Where does this plentitude of glycosyltransferases come from? Eukaryotes and within them green algae are not known to engage mobile genetic elements such as plasmids or, possibly, viruses that might transfer biosynthetic capabilities between strains as is common in bacteria. At present, no such mechanism for extra-chromosomal gene transfer in algae are known. The clear co-clustering of glycosylation traits with rRNA sequences [30] rather suggests a chromosomal location of the relevant glycosyltransferases. This, however, would entail that the different glycosyltransferases are products of evolutionary processes. In appreciation of the fact that land plants were not able to develop even a single glyco-enzyme in addition to those mosses had already contributed to the embryophyte taxon, the occurrence of gain-of-function leaps within what is considered a genus or clade, is—cautiously put—remarkable. In other words, we strongly suggest utilizing N-glycan profiles and structures as a relevant criterion for microalgae classification.

A note of caution: the experimental strategy used here, which involves pepsin digestion at low pH and MALDI-TOF MS of underivatized glycans, is sub-optimal for the detection of sialylated N-glycans as observed with human immunoglobulin and transferrin in the authors lab. However, sialylated species were likewise not detected when preparations were conducted at neutral pH and with MALDI-TOF MS analysis of permethylated N-glycans. Likewise, ESI-MS of glycopeptides did not detect any sialylated species in other microalgae [38,39,40], nor did other researchers detect sialic acids in algae [40,41,42].

At this point, we should admit that the research presented herein can hardly be seen as complete as more live strains could be grown and analyzed, more products could be ordered and re-ordered, more LC-MS runs could be performed, more DNA barcoding on more loci could be attempted or alternative genetic analyses could be applied. We think, however, that the value of raising awareness of the recently discovered possibility of characterizing microalgae strains by their N-glycans outweighs any limitations that could not be curtailed in a reasonable time span with the resources available. The conclusion is already clear that glycan patterns can serve to identify the origin, identity and purity of microalgal strains and products. While the currently applied procedure requires considerable time and work power, more efficient processes could be applied to control algae in a manner similar to the analysis of fish authenticity by MALDI-TOF MS [43].

It is important to emphasize that by no means do we want to question the presumed health benefits of the products regardless of the particular *Chlorella*-clade strain used. This work is not meant to undermine the reputation of any vendor or producer of algae as all the products can be expected to meet the specifications regarding vitamin and fatty acid content or other quality-determining features. Instead, this work is intended to stimulate broad interest in glycan-based strain characterization. We suppose that its application could benefit the food additive market as well as the manifold technical applications of microalgae strains, as in both areas the true identity of the used strains seems to be obscured by inappropriate, ambiguous species designations.

We hope and recommend that producers and taxonomists will recognize N-glycans as a valuable if not indispensable element in the toolbox for microalgae classification and identification.

## Figures and Tables

**Table 1:** Glyco-patterns of *Chlorella* products. N-glycan patterns generated by MALDI-TOF MS were grouped according to various features to the best of the authors’ abilities. Example spectra of glyco-patterns are found in Figure S2, all other spectra in Figure S6. Glyco-patterns put in brackets were mentioned previously [30] but did not re-occur in new samples. The normalized “virtual” retention times (v-min) apply to MH^+^ ions of reduced glycans with *m*/*z* values as given in parenthesis.

Man9 Group (Figure 4)	High Mass Pattern (Figure 6)	Dominant Mass (M+Na]^+^	Glyco-Pattern /Specific Features	Product Number (Table S1)	PGC-LC v-min (*m/z*)
1	weak l or l	1401	Hel	C-32, 33, 40, 65, 112, 121, 130, 156	>40 (1381)
1	l	1049	Raa	C-6, 95, 98, 110, 165, 178	17.3 (1029)
3	l	1049	Raa	C-55, 60, 63, 64a, 64b, 68, 70, 76, 133, 158, 159, 160, 180	17.3 (1029)
4	weak l or l	1049	Raa	C-96, 102, 134, 138, 143, 148, 162, 168, 170	17.3 (1029)
6	s	1049	Raa	C-141, 149	17.3 (1029)
6	t	1049	Raa	C-135, 154, 179	17.3 (1029)
1	l	no	Sol	C-3, 4, 8, 10, 21, 22,39a, 39b, 47, 53, 69, 88, 113, 117, 128, 142, 144, 145, 146	32.9 (1309)
2	weak l or l	no	Jar	C-2, 7, 20, 45, 51, 52a, 52b, 61, 66, 72, 75, 78, 85, 87, 90, 91, 93, 94, 100, 103, 104, 105, 106, 118, 119, 129, 137, 147, 163	15.8 (1309)
2	m	no	Jar	C-58	15.8 (1309)
5	weak m or m	no	Gov	C-13, 30, 35, 43, 44, 56a, 56b, 62, 97, 101	15.8 (1309)
7	weak m or m	no	Gov	C-38, 41	15.8 (1309)
4	m	no	Asp	C-25, 57, 59, 71, 73, 74, 77, 81, 109, 111	15.8 (1309)
5	l	no	Vul	C-80, 126	
5	m	no	Vul	C-26, 37, 42, 131	
5	o	no	Vul	C-9, 11, 27, 31	
3	none	no	Vul	C-86	
6	r	no	Vul	C-114	
1	l	1343	Kei	C-1, 54, 115, 151	33.0 (1323)
5	l	1049	(Now)	C-5	
10	weak m	1063		C-173	
10	u	1063		C-174	
10	none	1063		C-175	
5	none	1107		C-116	
5	none	1255	(Pit)	C-15, 17	
6	q	1269		C-46	
5	l	1296	(Sun)	C-16	
4	l	1296	(Sun)	C-36	
2	none	1299	x	C-122	
1	l	1343/1401	Mix Kei + Hel	C-108 (MALDI MS2 as for “Kei” and “Hel”)	>40 (1381)
1	p	1401	>2 HexNAcs	C-153 (MS2 of 1401 ≈ “Hel”)	
1	weak l or l	1373	(Jos)	C-23, 24, 29	
1	l	1637		C-132, 161, 166	
1	none	no		C-14	
1	l	no		C-89 (MS2 of 1343 ≈ “Sol”	32.9 (1309)
2	none	no	1211 unique	C-79 (MS2 of *m/z* = 1211 unique)	
3	l	no	1079, 1389	C-67	
4	ca. m	no	boring	C-92	
4	m	no	1211 unique	C-120 (MS2 of *m/z* = 1211 unique)	
5	ca. o	no	>2 HexNAcs,	C-28	
5	o	no	1269, 1607	C-82, 164 (MS2 of1 1269 unlike C-46)	
6	q	no		C-152	
7	p	no	>2 HexNAcs	C-127	
7	q	no	very crowded	C-125	
7	v	no	1107, 1989	C-136 (MS2 of 1107 as for C-80 and C-116)	
7	m	no	1195, 1683	C-139	
7	l	no	1310	C-140	
7	l	no	1343, 1898	C-150	
7	none	no	1049	C-157	
7	weak m	no	1371	C-167	
8	none	no	1211, 1373	C-99	
8	l	no		C-155 (MS2 of *m/z* = 1211 ≈ “Sol”)	32.9 (1309)
8	l	no		C-171 (MS2 of *m/z* = 1343 ≈ “Sol”)	32.9 (1309)
8	weak m	no	1533, 1575	C-176	
8	none	no	1389	C-177	
9	none	no	>2 HexNAcs	C-172	

## Data Availability

The data presented in this study are available on request from the corresponding author (accurately indicate status).

## Supplementary Materials

The following supporting information can be downloaded at: https://www.mdpi.com/article/10.3390/foods13193182/s1, File “Supporting Figures” with extra parts Supporting Figures S2 and S6. File “Supporting Sequences”. File “*Chlorella* products Supporting Tables.pdf“ with extra part “Supporting Tables.xls”.

## Abbreviations

A^4^A^4^F^6^	abbreviation for a biantennary, fucosylated N-glycan according to the proglycan scheme [37].
^13^C_4_-A^4^A^4^F^6^	isotope-labeled N-glycan with four ^13^C-containing N-acetyl groups;
“Hel”: “Raa”, “Sol”, “Jar”, “Gov”, “Asp”, “Vul”, “Kei”,	designations of N-glycan patterns;
HexNAc	*N*-acetylhexosamines;
LIF	laser-induced fragmentation;
MALDI-TOF MS	Matrix-assisted ionization time-of-flight mass spectrometry;
OM	oligomannosidic.
PGC-LC-MS	liquid chromatography electrospray-ionization mass spectrometry on porous graphitic carbon;
PNGase A	peptide: N-glycosidase A;
