# Supplementary material for: The Diversity of N-Glycans of Chlorella Food Supplements Challenges Current Species Classification
Source: Foods. 2024 Oct 7;13(19):3182. doi: 10.3390/foods13193182 (PMC11482596; doi:10.3390/foods13193182)
Supplement: Supplementary file 1 [file foods-13-03182-s001.zip › Chlorella Products Supporting Figures Foods.pdf]

Supporting Figures 1, 3-5, 7 and 8 for

The diversity of N-glycans of *Chlorella* food supplements challenges current species classification

Réka Mócsai [1], Johannes Helm [1], Karin Polacsek [1], Johannes Stadlmann [1], Friedrich Altmann [1,*]

Department of Chemistry, BOKU University, Muthgasse 18, Vienna, Austria
rekamocsai@gmail.com (R.M.); jhelm@gmx.net (J.H.); karin.polacsek@boku.ac.at (K.P.); j.stadlmann@boku.ac.at (J.S.); friedrich.altmann@boku.ac.at (F.A.)
* Correspondence: friedrich.altmann@boku.ac.at)

Figure S1 MALDI-TOF MS spectra of live algae strains representing *C. vulgaris* and two different types of *C. sorokiniana.*

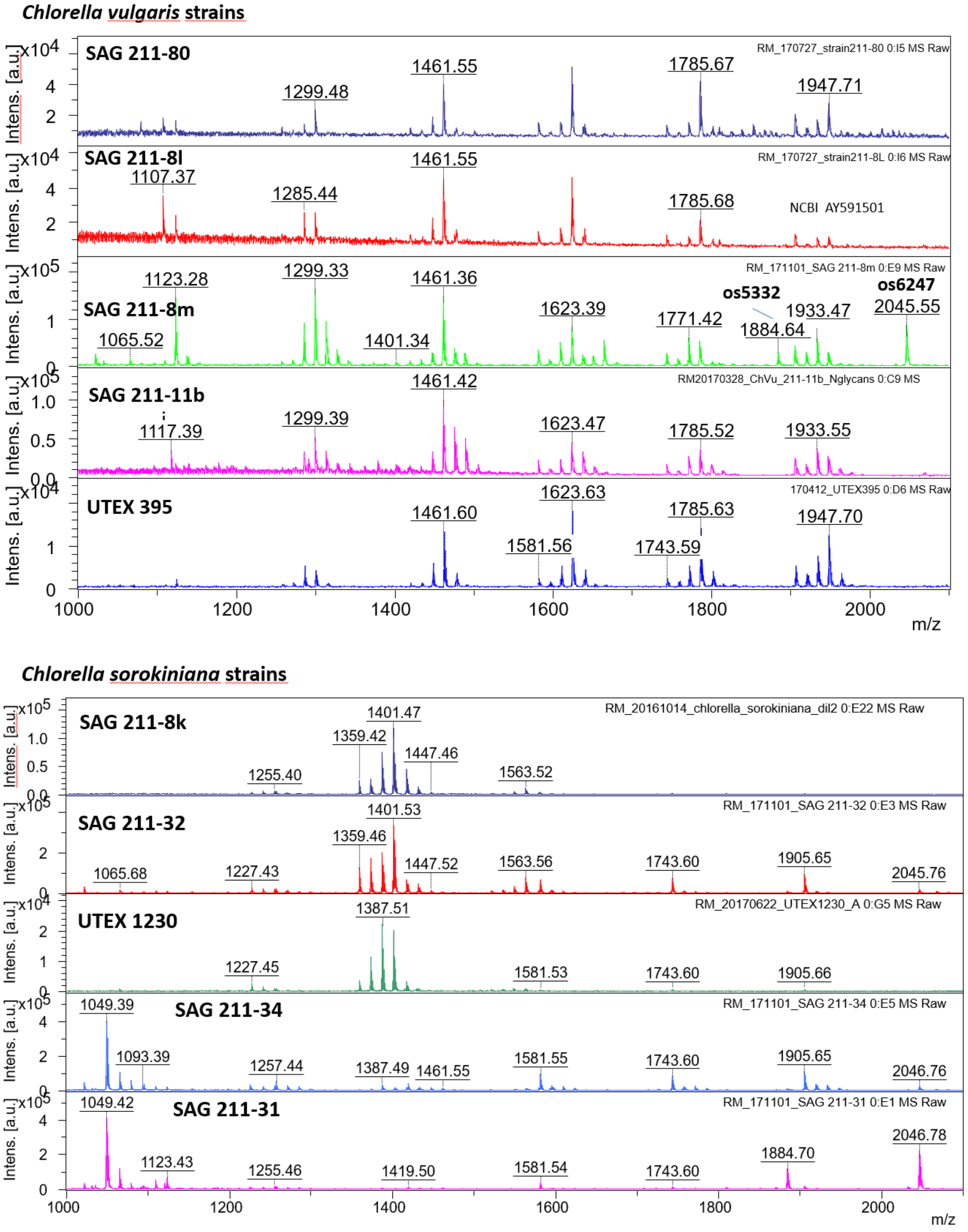

Figure S2: Exemplary spectra of glyco-groups of *Chlorella* products. Order essentially as in Table 1.

(5 pages – extra PDF file)

Figure S3 MALDI-TOF MS/MS spectra of m/z =1049 (os2221) in selected samples. The upper two examples are representative for all samples with a "Raa" glycan pattern irrespective of oligomannose methylation. The bottom spectrum – though similar was derived from a different N-glycan structure [1]. Fragment ions are annotated with their numbers of hexose, HexNAc, pentose and methyl constituents. As – in this case – the structures have been determined, fragment cartoons are given here. By the way, this shows that interpretation of fragment ions requires prior knowledge of the structure.

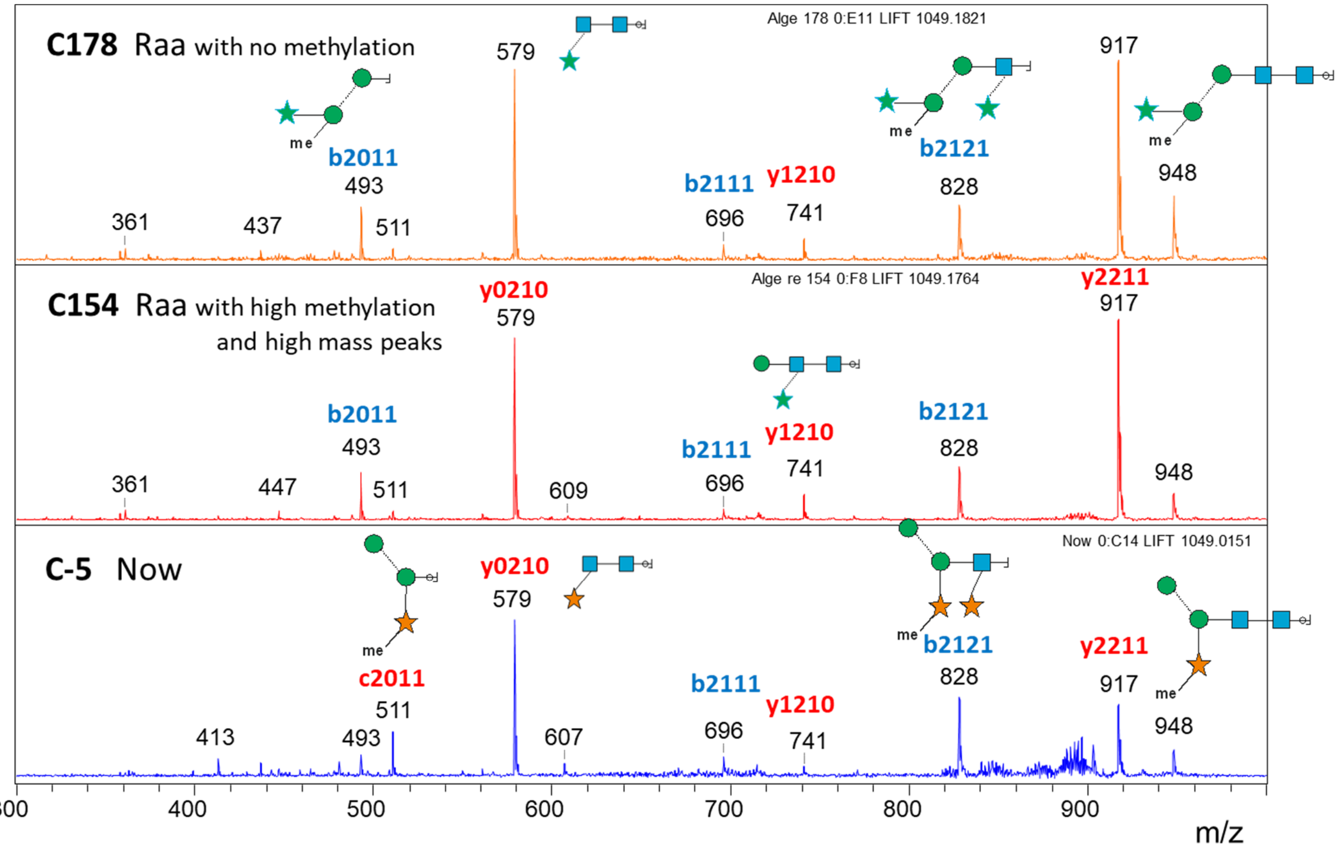

Figure S4 Scrutiny of os2221 from different "Raa"-type samples by PGC-LC-MS. Panel **A** shows the MALDI-TOF MS spectra of the selected examples. Panel **B** depicts the XICs of the respective [M+H]+ ion together with brain N-glycans that served as internal standard. The peaks with the correct mass are indicated by the blue arrow. All other peaks had differing masses.

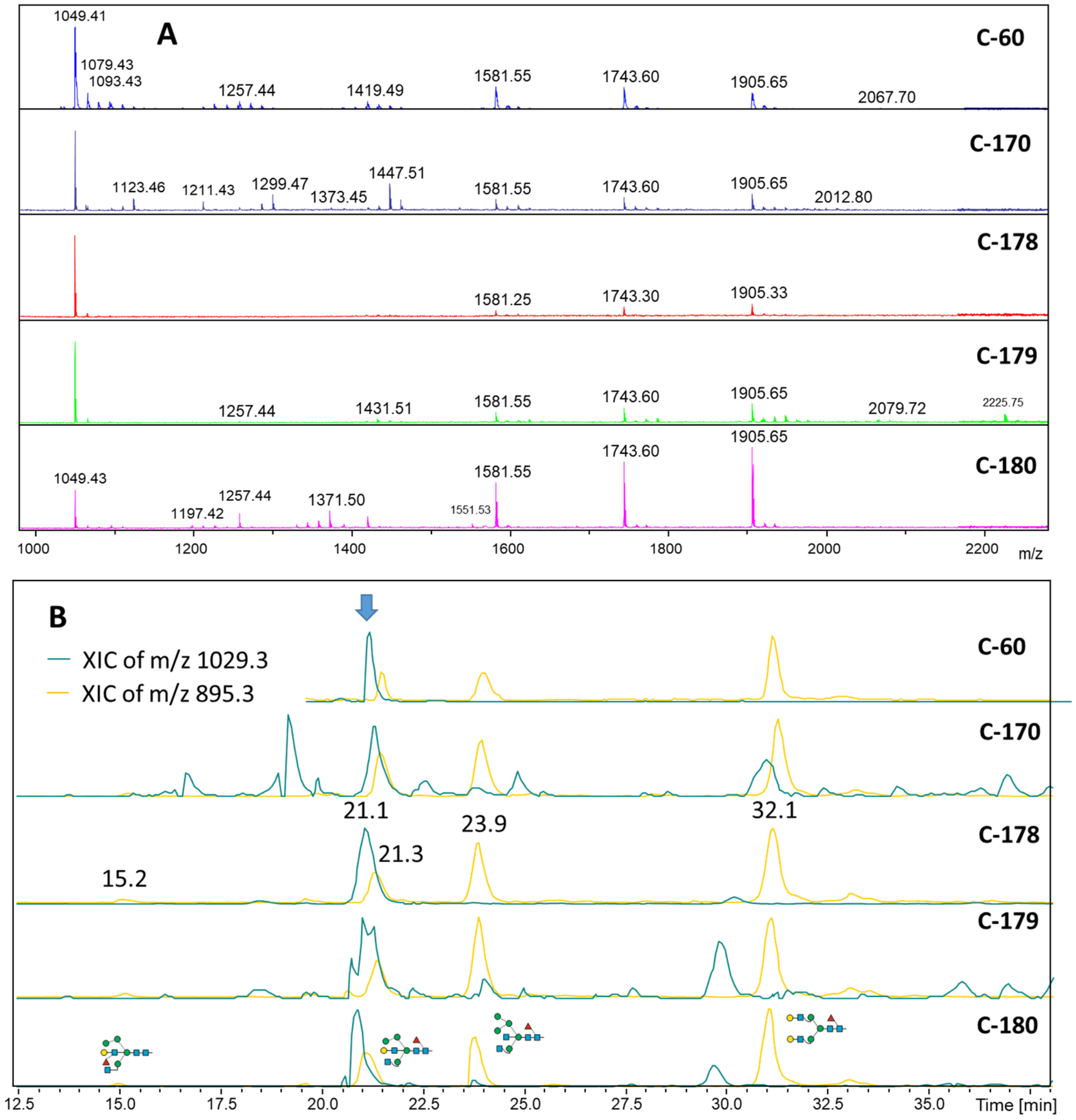

Figure S5 Comparison of three m/z = 1269 peaks by MALDI-TOF MS/MS. Samples C-46 and C-152 apparently contain a substituted (maybe fucose) reducing GlcNAc, while the same peak in C-82 has a clearly different structure.

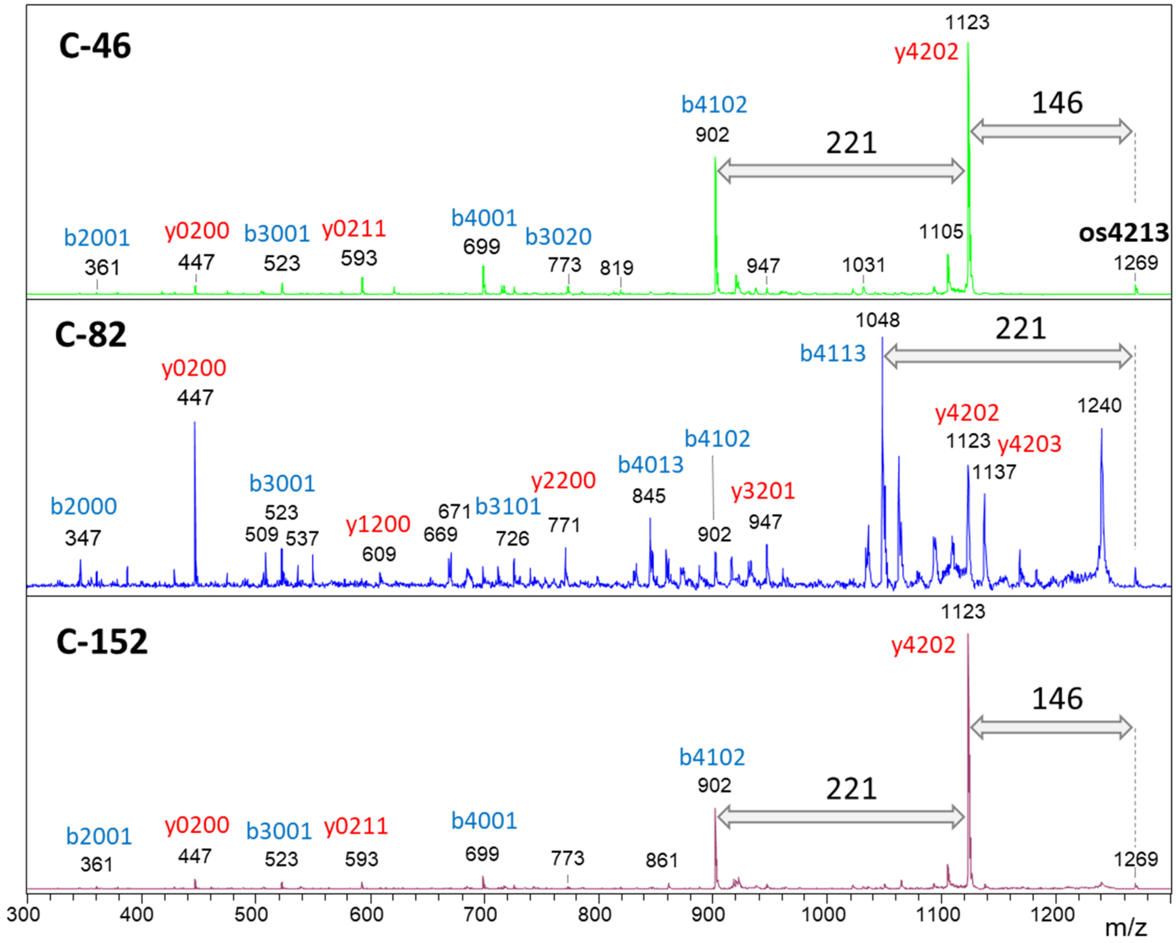

Figure S6 Spectra of ungrouped Chlorella products. Order as in Table 1.

(8 pages -- see separate PDF file)

Figure S7 Comparison of sample C-108, C-1 and C-32 by MALDI-TOF MS and PGC-LC-MS and MS/MS with emphasis on the MALDI-TOF MS peaks with *m/z* = 1343 (os3231) and 1401 (os4223). Panel **A** gives the MALDI-TOF MS spectra. Panel **B** shows the elution on PGC of os4223 from C-32 and C108 supported by internal standards (brain glycans). Panel **C** shows a similar comparison for os3231 and panel **D** presents the associated MS/MS spectra. Panel **E** demonstrates the difference of spectra for *m/z* = 1323 in sample C-108.

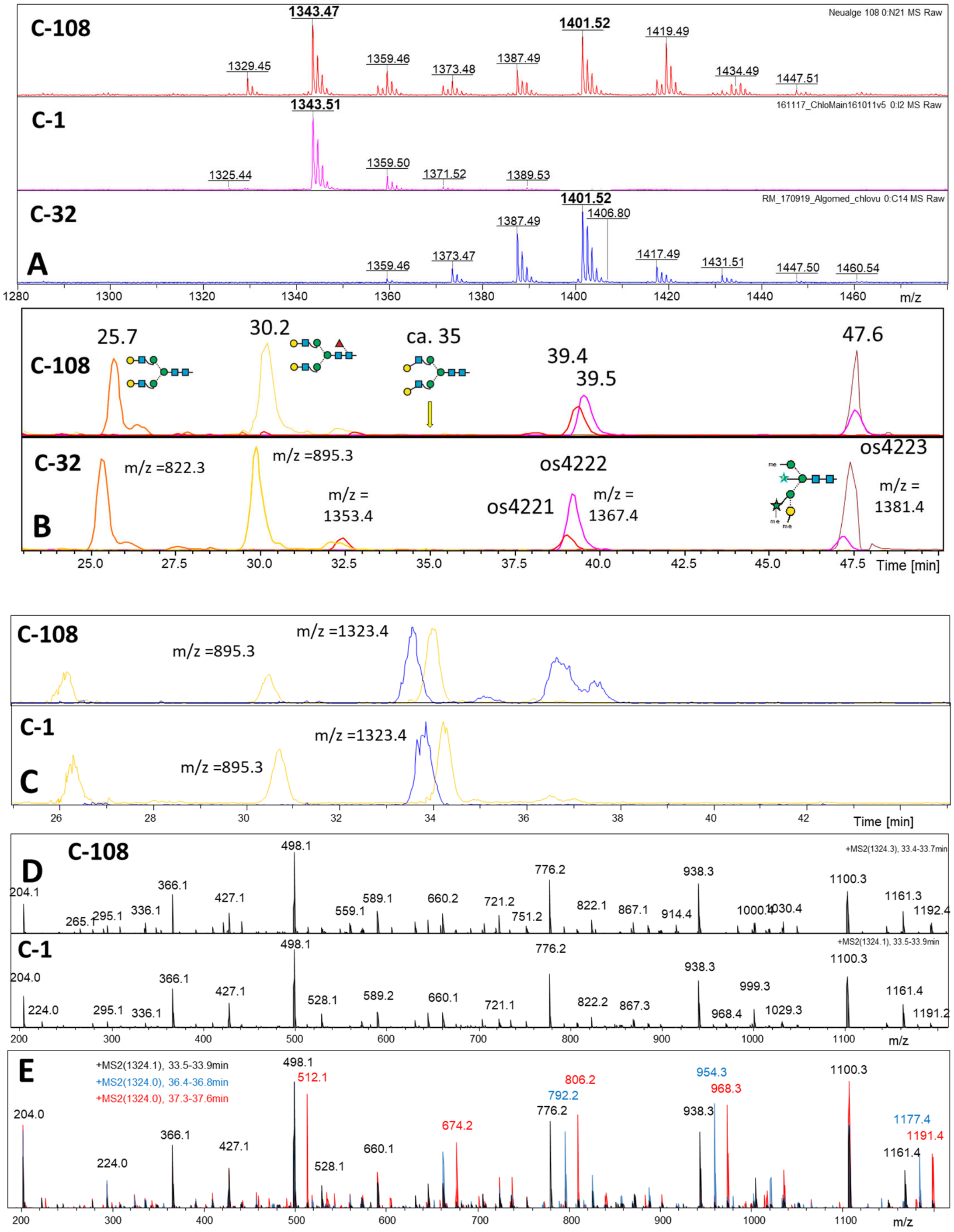

Figure S8 Identities and deviations of nucleotide sequences of the ITS1-5.8S-ITS2 region as calculated with web.expasy.org/cgi-bin/sim. Considered that a) the more highly conserved 5.8S rDNA accounts of more than a quarter of the sequence and that b) a totally unrelated sequence as *e.g.*, bovine albumin yield "identities" of over 40 %. That means that the yellow and green cells indicate essentially unrelated sequences.

	**211-11b**	**Hel**	**Raa**	**Sol**	**Jar**	**Gov**	Kei	Jos	Ori28	Ori46A	Ori46B	Ori46C	SunA	SunB	
	**% identity / Deviation of sequences between TTTCCGTAGGTG... and ...TTCGACCT**														
**211-11b**	100	24.7	26.1	25	25.7	31	24.6	25	31.2	31.2	24.6	37.4	24.9	38.8	**Deviation**
**Hel**	75.3	100	23.7	9.3	9.1	28	6.1	7.3	26.8	26.2	9.8	36.4	23.7	36.8	
**Raa**	73.9	76.3	100	24.9	23.7	28.5	22.2	22	27.3	26.2	23.4	36.8	16.8	37.1	
**Sol**	75	90.7	75.1	100	9.6	26.7	8.8	10	26.6	26.1	8.7	36.1	24.2	36.9	
**Jar**	74.3	90.9	76.3	**90.4**	100	27.2	6.1	5.7	25.9	25.7	10.9	36.4	23	36.7	
**Gov**	69	72	71.5	**73.3**	**72.8**	100	27.7	28.8	15.8	14.8	26.6	34.1	26.8	35.3	
Kei	75.4	93.9	77.8	91.2	93.9	72.3	100	3.1	26.1	26.2	9.7	35.1	22.5	36.2	
Jos	75	92.7	78	90	94.3	71.2	96.9	100	26.2	25.8	10.7	35.4	22	36.9	
Ori28	68.8	73.2	72.7	73.4	74.1	84.2	73.9	73.8	100	16	25.3	35.8	25.4	37.5	
Ori46A	68.8	73.8	73.8	73.9	74.3	85.2	73.8	74.2	84	100	25.6	35.5	24.6	35.9	
Ori46B	75.4	90.2	76.6	91.3	89.1	73.4	90.3	89.3	74.7	74.4	100	35.8	25.4	36.5	
Ori46C	62.6	63.6	63.2	63.9	63.6	65.9	64.9	64.6	64.2	64.5	64.2	100	35	3.9	
SunA	75.1	76.3	83.2	75.8	77	73.2	77.5	78	74.6	75.4	74.6	65	100	35.3	
SunB	61.2	63.2	62.9	63.1	63.3	64.7	63.8	63.1	62.5	64.1	63.5	96.1	64.7	100	
	**Identity**														

References

1. Mocsai, R.; Kaehlig, H.; Blaukopf, M.; Stadlmann, J.; Kosma, P.; Altmann, F. The Structural Difference of Isobaric N-Glycans of Two Microalgae Samples Reveals Taxonomic Distance. *Front Plant Sci* **2021**, *12*, 643249, https://doi.org/10.3389/fpls.2021.643249.
