# Supplementary material for: The Diversity of N-Glycans of Chlorella Food Supplements Challenges Current Species Classification
Source: Foods. 2024 Oct 7;13(19):3182. doi: 10.3390/foods13193182 (PMC11482596; doi:10.3390/foods13193182)
Supplement: Supplementary file 1 [file foods-13-03182-s001.zip › Figure S2 Chlorella products spectra grouped.pptx]

## Slide 1
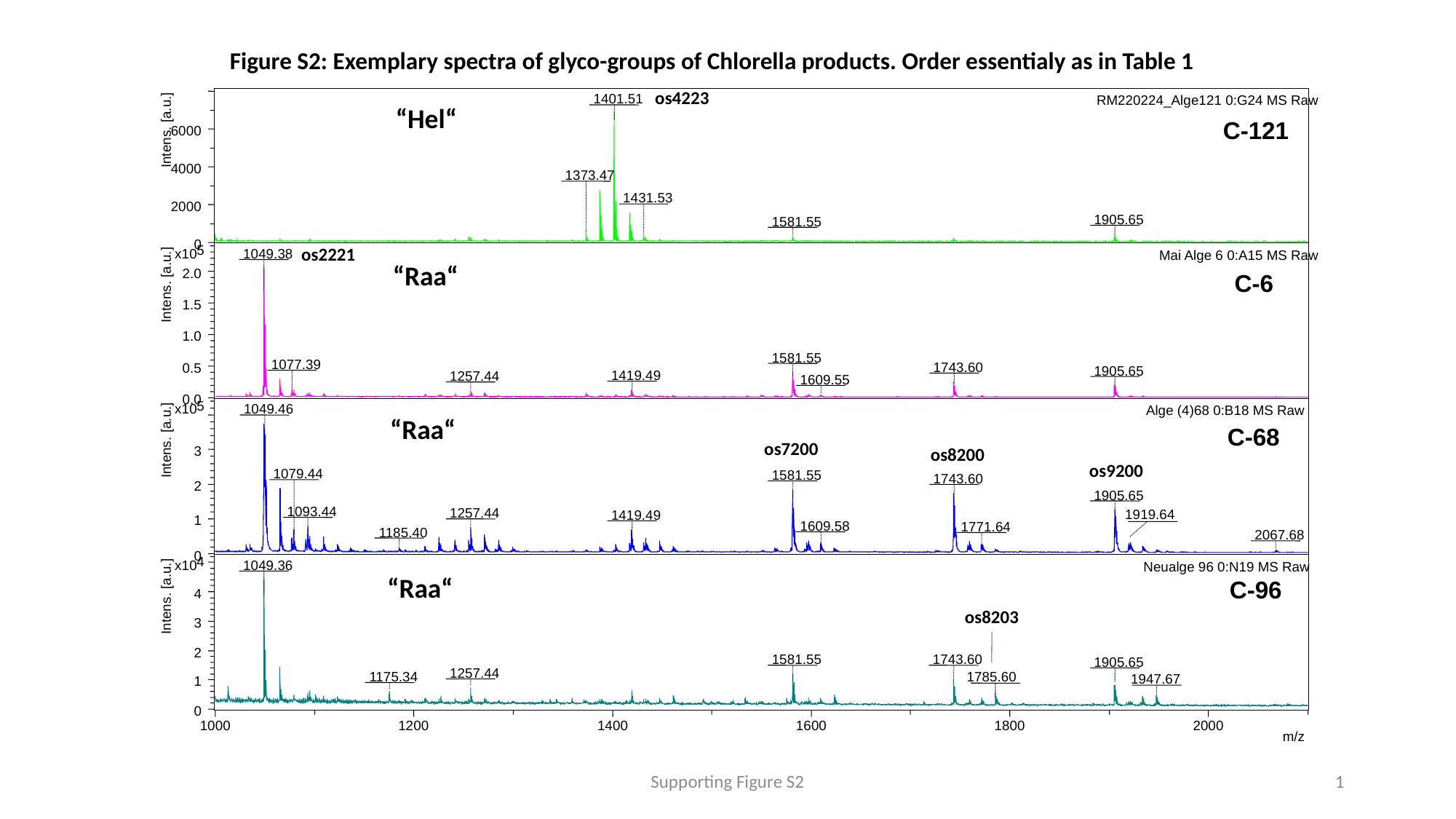

Figure S2: Exemplary spectra of glyco-groups of Chlorella products. Order essentialy as in Table 1
os4223
1401.51
RM220224_Alge121 0:G24 MS Raw
Intens. [a.u.]
6000
4000
1373.47
1431.53
2000
1905.65
1581.55
0
5
x10
1049.38
Mai Alge 6 0:A15 MS Raw
2.0
Intens. [a.u.]
1.5
1.0
1581.55
1077.39
1743.60
0.5
1905.65
1419.49
1257.44
1609.55
0.0
5
x10
1049.46
Alge (4)68 0:B18 MS Raw
Intens. [a.u.]
3
1079.44
1581.55
1743.60
2
1905.65
1093.44
1257.44
1419.49
1
1609.58
1771.64
1185.40
2067.68
0
1049.36
Neualge 96 0:N19 MS Raw
1581.55
1743.60
1905.65
1257.44
1175.34
1
0
“Hel“
C-121
os2221
“Raa“
C-6
“Raa“
C-68
os7200
os8200
os9200
1919.64
4
x10
“Raa“
C-96
4
Intens. [a.u.]
os8203
3
2
1785.60
1947.67
1000
1200
1400
1600
1800
2000
m/z
Supporting Figure S2
1

## Slide 2
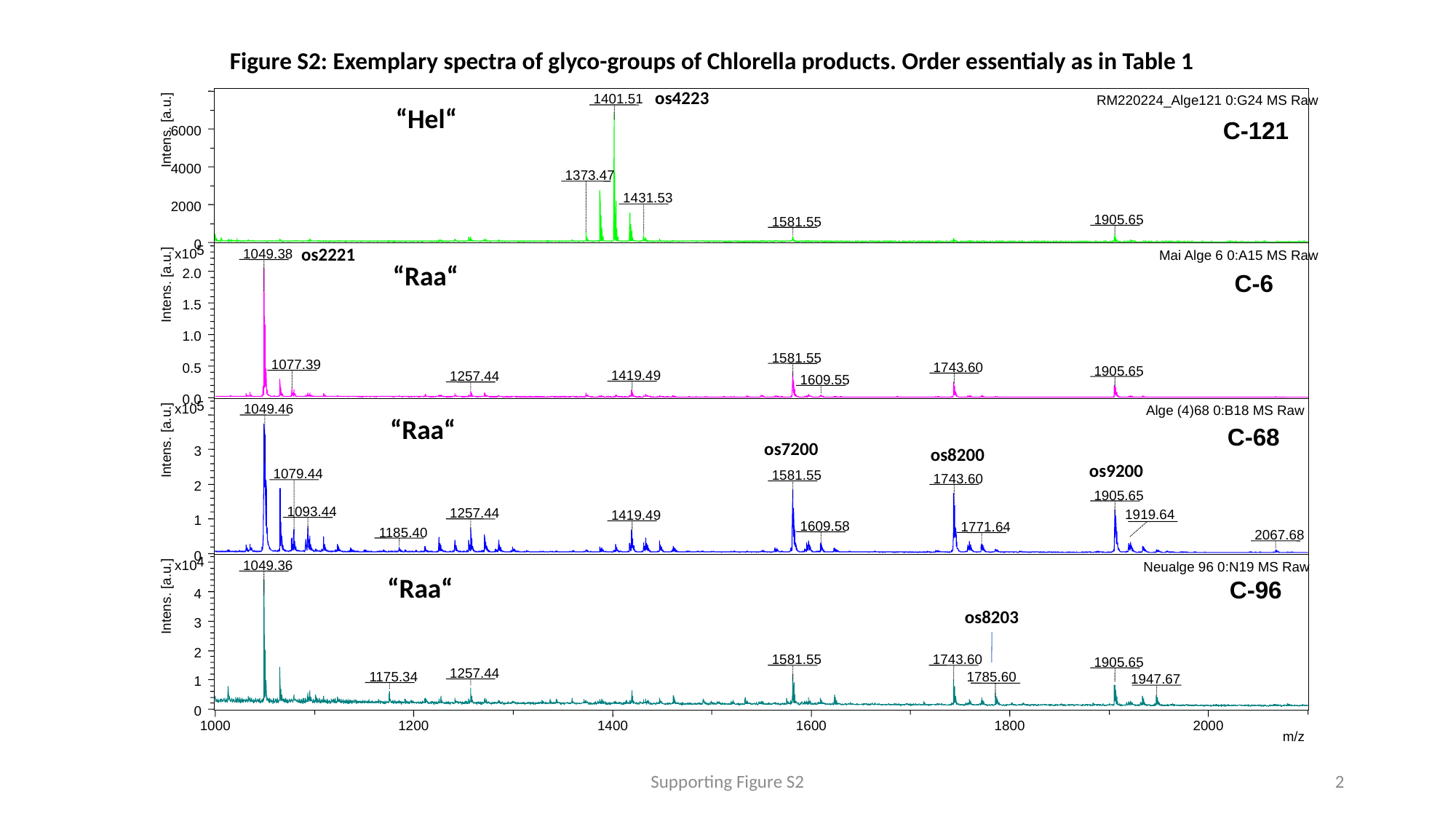

Figure S2: Exemplary spectra of glyco-groups of Chlorella products. Order essentialy as in Table 1
os4223
1401.51
RM220224_Alge121 0:G24 MS Raw
Intens. [a.u.]
6000
4000
1373.47
1431.53
2000
1905.65
1581.55
0
5
x10
1049.38
Mai Alge 6 0:A15 MS Raw
2.0
Intens. [a.u.]
1.5
1.0
1581.55
1077.39
1743.60
0.5
1905.65
1419.49
1257.44
1609.55
0.0
5
x10
1049.46
Alge (4)68 0:B18 MS Raw
Intens. [a.u.]
3
1079.44
1581.55
1743.60
2
1905.65
1093.44
1257.44
1419.49
1
1609.58
1771.64
1185.40
2067.68
0
1049.36
Neualge 96 0:N19 MS Raw
1581.55
1743.60
1905.65
1257.44
1175.34
1
0
“Hel“
C-121
os2221
“Raa“
C-6
“Raa“
C-68
os7200
os8200
os9200
1919.64
4
x10
“Raa“
C-96
4
Intens. [a.u.]
os8203
3
2
1785.60
1947.67
1000
1200
1400
1600
1800
2000
m/z
Supporting Figure S2
2

## Slide 3
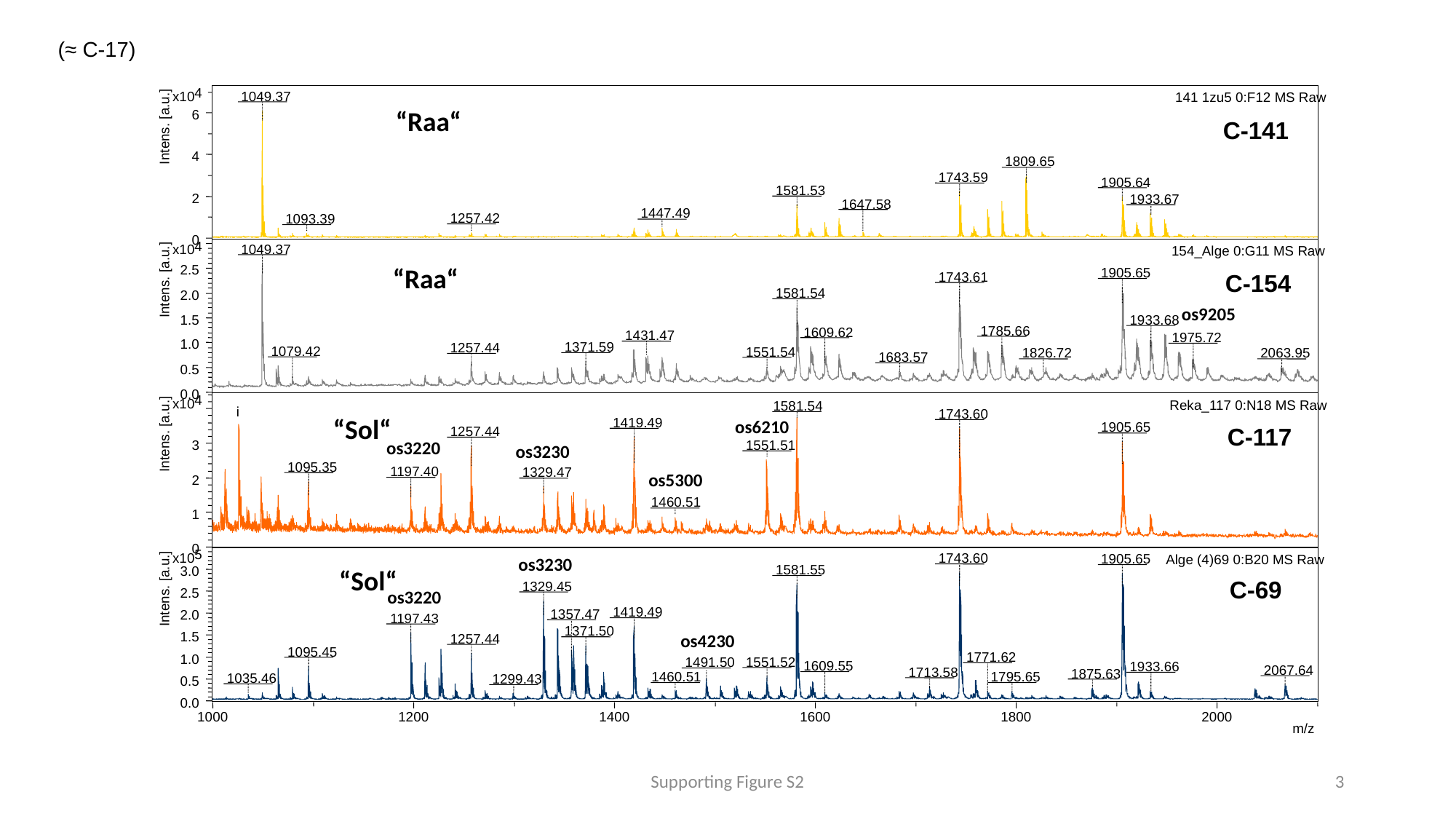

(≈ C-17)
4
x10
1049.37
141 1zu5 0:F12 MS Raw
“Raa“
6
C-141
Intens. [a.u.]
4
1809.65
1743.59
1905.64
1581.53
2
1933.67
1647.58
1447.49
1257.42
1093.39
0
4
1049.37
x10
154_Alge 0:G11 MS Raw
“Raa“
2.5
C-154
1905.65
1743.61
Intens. [a.u.]
1581.54
2.0
os9205
1.5
1933.68
1785.66
1609.62
1431.47
1975.72
1.0
1371.59
1257.44
1079.42
1551.54
1826.72
2063.95
1683.57
0.5
0.0
4
x10
Reka_117 0:N18 MS Raw
1581.54
i
1743.60
“Sol“
os6210
1419.49
C-117
1905.65
1257.44
Intens. [a.u.]
os3220
os3230
3
1551.51
1095.35
1197.40
os5300
1329.47
2
1460.51
1
0
5
os3230
x10
1743.60
1905.65
Alge (4)69 0:B20 MS Raw
“Sol“
1581.55
3.0
C-69
1329.45
Intens. [a.u.]
os3220
2.5
1419.49
1357.47
2.0
1197.43
1371.50
os4230
1.5
1257.44
1095.45
1771.62
1.0
1551.52
1491.50
1609.55
1933.66
2067.64
1713.58
1875.63
1460.51
1795.65
1035.46
1299.43
0.5
0.0
1000
1200
1400
1600
1800
2000
m/z
Supporting Figure S2
3

## Slide 4
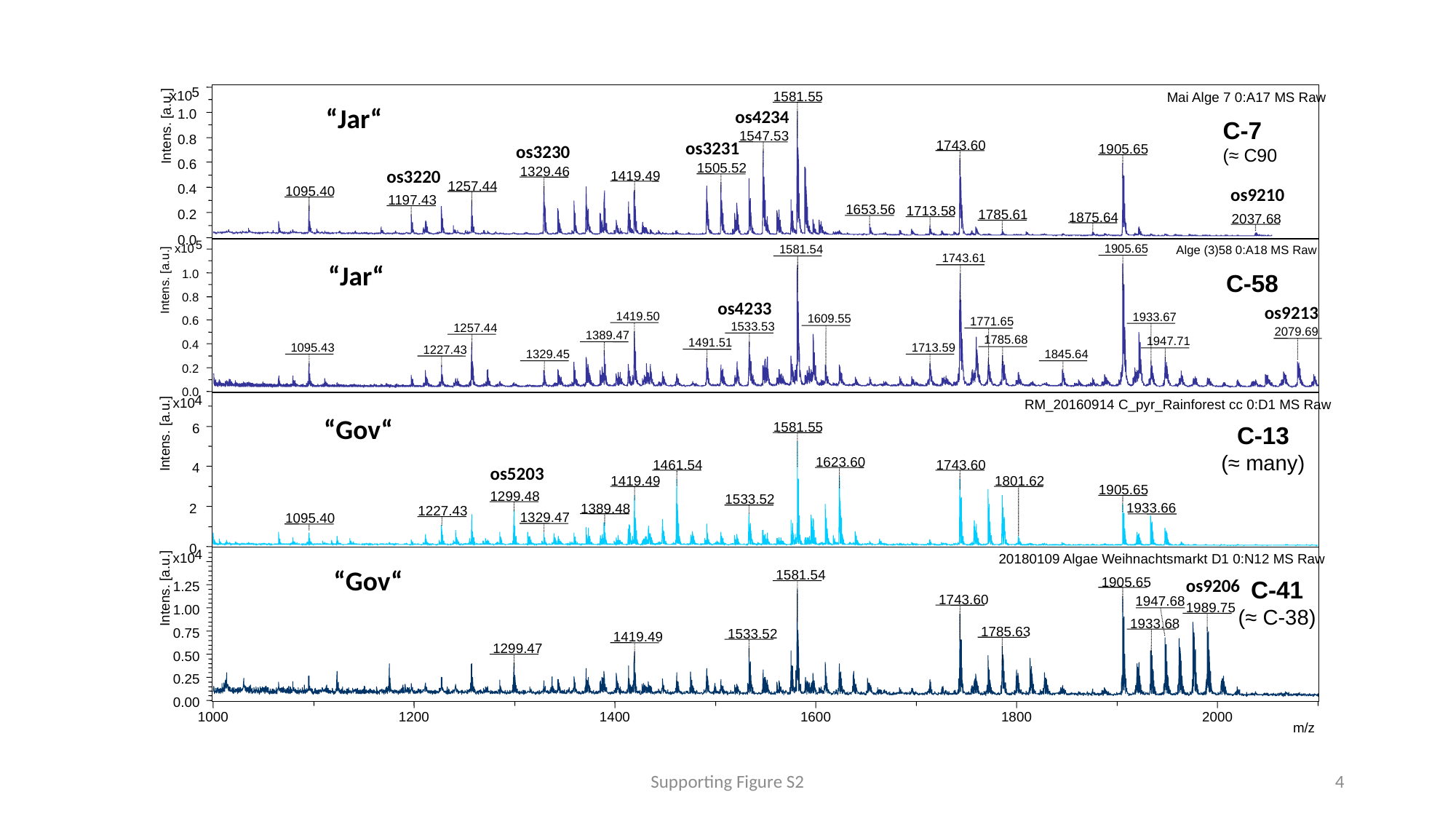

5
x10
1.0
Intens. [a.u.]
0.8
0.6
0.4
0.2
0.0
1581.55
Mai Alge 7 0:A17 MS Raw
“Jar“
os4234
C-7
(≈ C90
1547.53
os3231
os3230
1743.60
1905.65
1505.52
os3220
1329.46
1419.49
1257.44
os9210
1095.40
1197.43
1653.56
1713.58
1785.61
1875.64
2037.68
5
x10
1905.65
1581.54
Alge (3)58 0:A18 MS Raw
1743.61
1.0
Intens. [a.u.]
0.8
1419.50
1933.67
1609.55
0.6
1771.65
1533.53
1257.44
2079.69
1389.47
1785.68
1947.71
1491.51
0.4
1095.43
1713.59
1227.43
1329.45
1845.64
0.2
0.0
“Jar“
C-58
os4233
os9213
4
x10
RM_20160914 C_pyr_Rainforest cc 0:D1 MS Raw
“Gov“
C-13
(≈ many)
1581.55
6
Intens. [a.u.]
1623.60
1461.54
1743.60
os5203
4
1419.49
1801.62
1905.65
1299.48
1533.52
1933.66
2
1389.48
1227.43
1329.47
1095.40
0
4
x10
20180109 Algae Weihnachtsmarkt D1 0:N12 MS Raw
“Gov“
1581.54
os9206
C-41
(≈ C-38)
1905.65
1.25
Intens. [a.u.]
1947.68
1743.60
1989.75
1.00
1933.68
1785.63
0.75
1533.52
1419.49
1299.47
0.50
0.25
0.00
1000
1200
1400
1600
1800
2000
m/z
Supporting Figure S2
4

## Slide 5
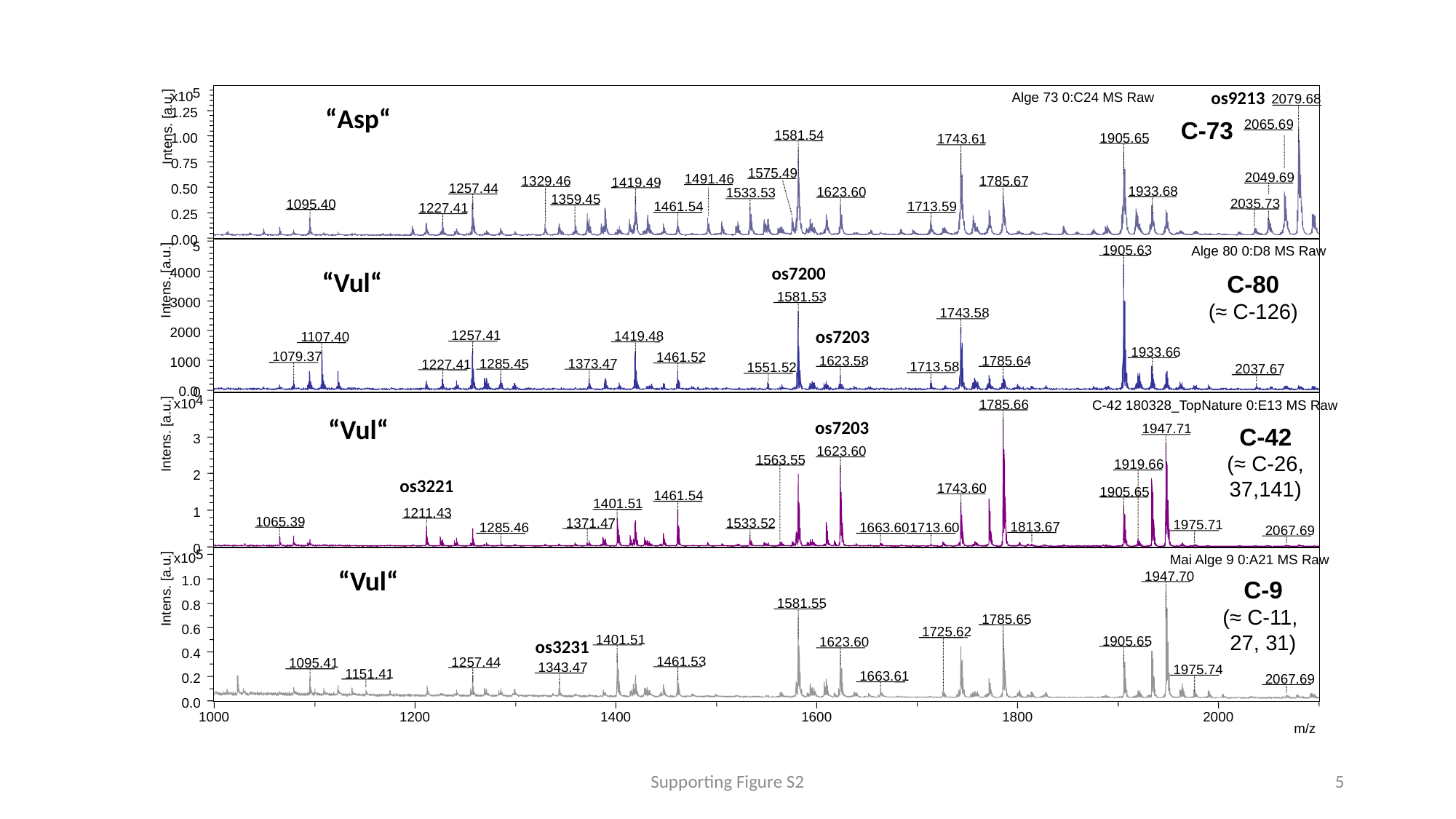

os9213
5
2079.68
x10
Alge 73 0:C24 MS Raw
“Asp“
1.25
C-73
2065.69
Intens. [a.u.]
1581.54
1.00
1905.65
1743.61
0.75
1575.49
2049.69
1491.46
1785.67
1329.46
1419.49
1257.44
0.50
1933.68
1623.60
1533.53
1359.45
2035.73
1095.40
1461.54
1713.59
1227.41
0.25
0.00
5
1905.63
Alge 80 0:D8 MS Raw
4000
Intens. [a.u.]
1581.53
3000
1743.58
2000
1257.41
1419.48
1107.40
1933.66
1079.37
1461.52
1623.58
1785.64
1000
1285.45
1373.47
1227.41
1713.58
1551.52
2037.67
0
os7200
“Vul“
C-80
(≈ C-126)
os7203
0.0
4
x10
1785.66
C-42 180328_TopNature 0:E13 MS Raw
“Vul“
os7203
C-42
(≈ C-26,
37,141)
1947.71
Intens. [a.u.]
3
1623.60
1563.55
1919.66
2
os3221
1743.60
1905.65
1461.54
1401.51
1
1211.43
1065.39
1371.47
1533.52
1975.71
1813.67
1285.46
1663.60
1713.60
2067.69
0
5
x10
Mai Alge 9 0:A21 MS Raw
“Vul“
1947.70
C-9
(≈ C-11,
27, 31)
1.0
Intens. [a.u.]
1581.55
0.8
1785.65
0.6
1725.62
os3231
1401.51
1905.65
1623.60
0.4
1461.53
1257.44
1095.41
1343.47
1975.74
1151.41
1663.61
0.2
2067.69
0.0
1000
1200
1400
1600
1800
2000
m/z
Supporting Figure S2
5

## Slide 6
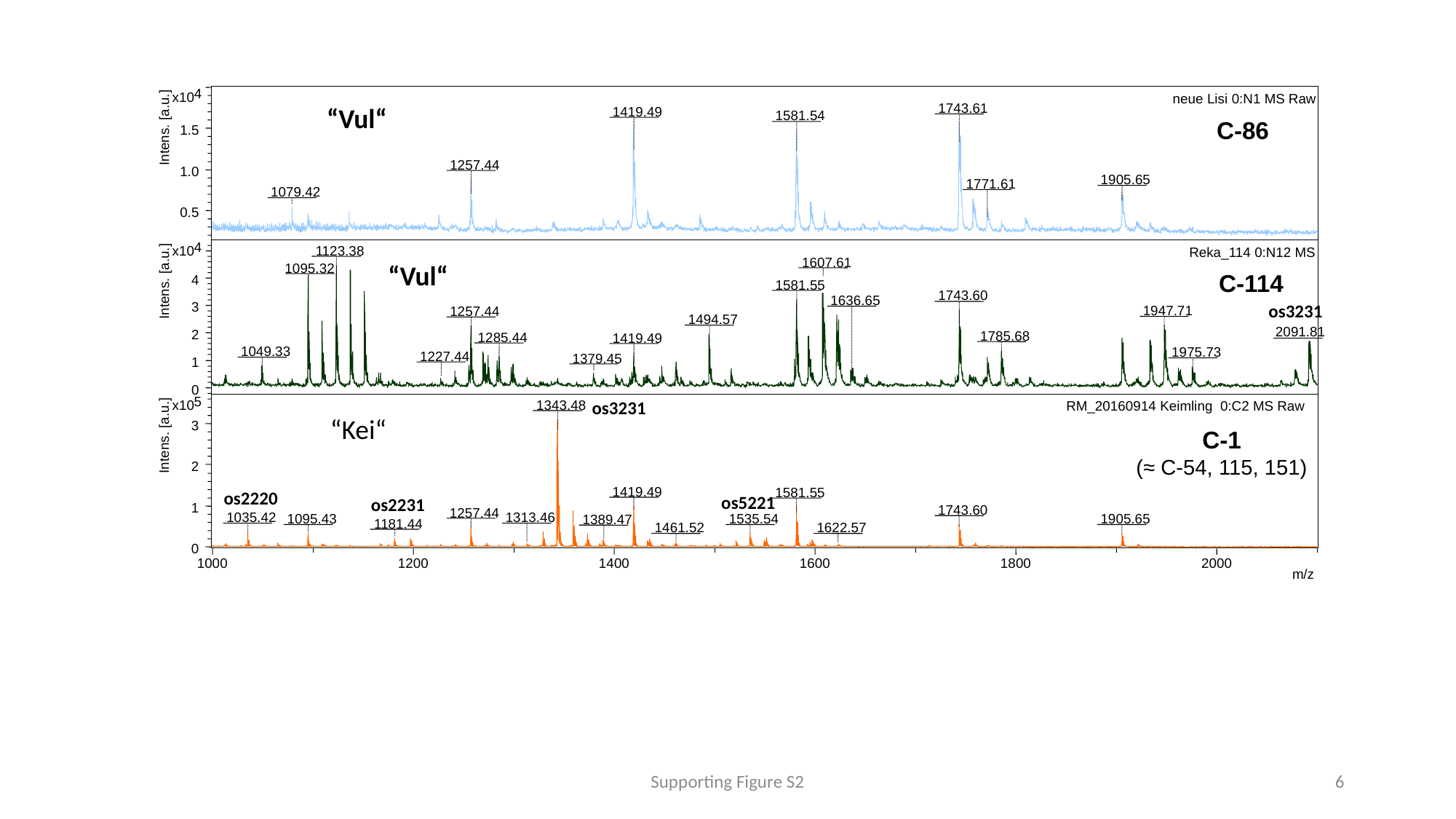

4
x10
neue Lisi 0:N1 MS Raw
“Vul“
1743.61
1419.49
1581.54
C-86
Intens. [a.u.]
1.5
1257.44
1.0
1905.65
1771.61
1079.42
0.5
4
x10
1123.38
Reka_114 0:N12 MS
“Vul“
1607.61
1095.32
C-114
4
Intens. [a.u.]
1581.55
1743.60
1636.65
os3231
3
1947.71
1257.44
1494.57
2091.81
2
1785.68
1285.44
1419.49
1049.33
1975.73
1227.44
1379.45
1
0
os3231
5
x10
1343.48
RM_20160914 Keimling 0:C2 MS Raw
“Kei“
3
C-1
(≈ C-54, 115, 151)
Intens. [a.u.]
2
os2220
1419.49
1581.55
os5221
os2231
1
1743.60
1257.44
1035.42
1313.46
1095.43
1535.54
1905.65
1389.47
1181.44
1461.52
1622.57
0
1000
1200
1400
1600
1800
2000
m/z
Supporting Figure S2
6
