# Supplementary material for: The Diversity of N-Glycans of Chlorella Food Supplements Challenges Current Species Classification
Source: Foods. 2024 Oct 7;13(19):3182. doi: 10.3390/foods13193182 (PMC11482596; doi:10.3390/foods13193182)
Supplement: Supplementary file 1 [file foods-13-03182-s001.zip › Figure S6 Chlorella products spectra unassigned.pptx]

## Slide 1
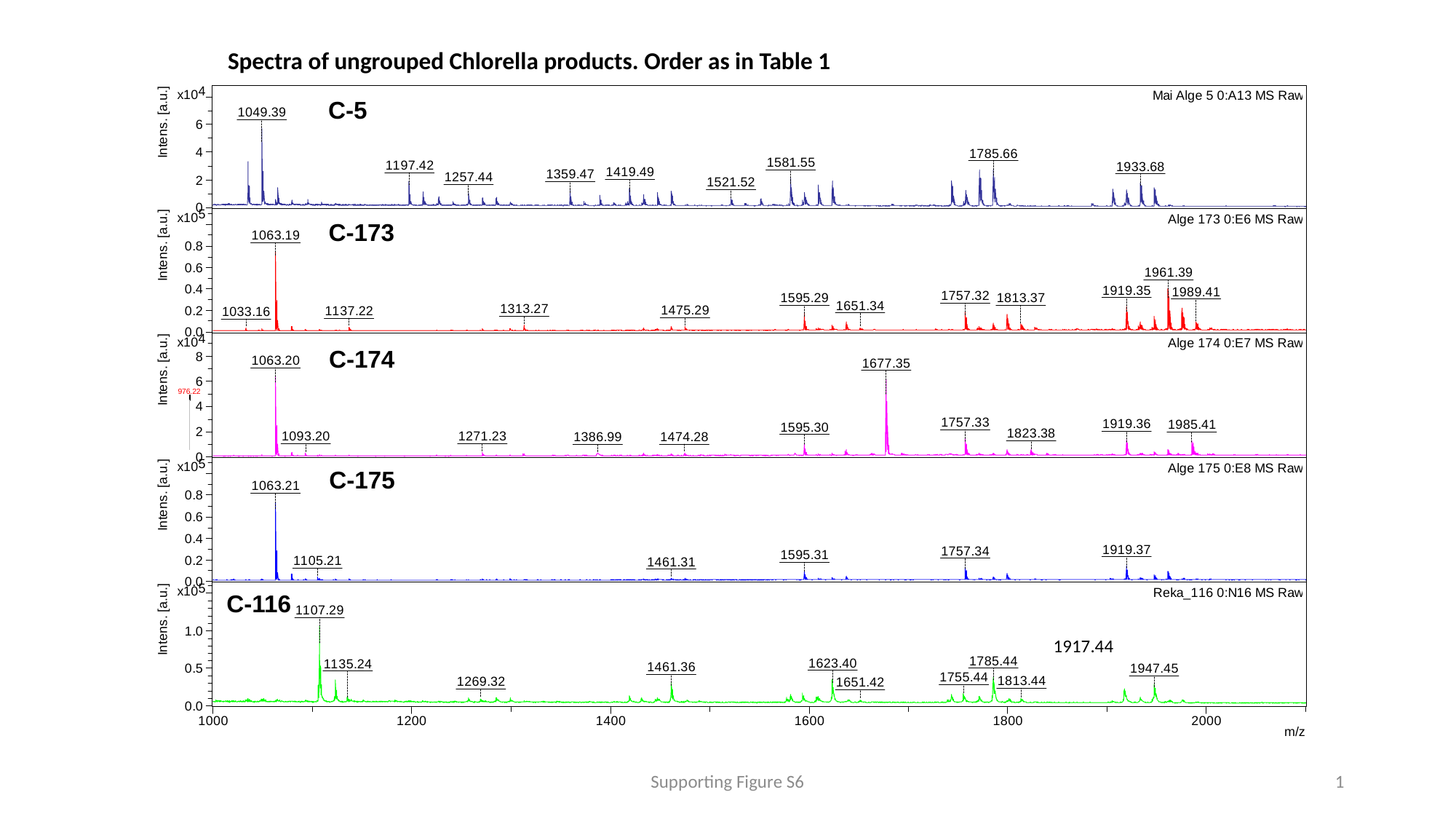

Spectra of ungrouped Chlorella products. Order as in Table 1
C-5
C-173
C-174
C-175
C-116
1917.44
Supporting Figure S6
1

## Slide 2
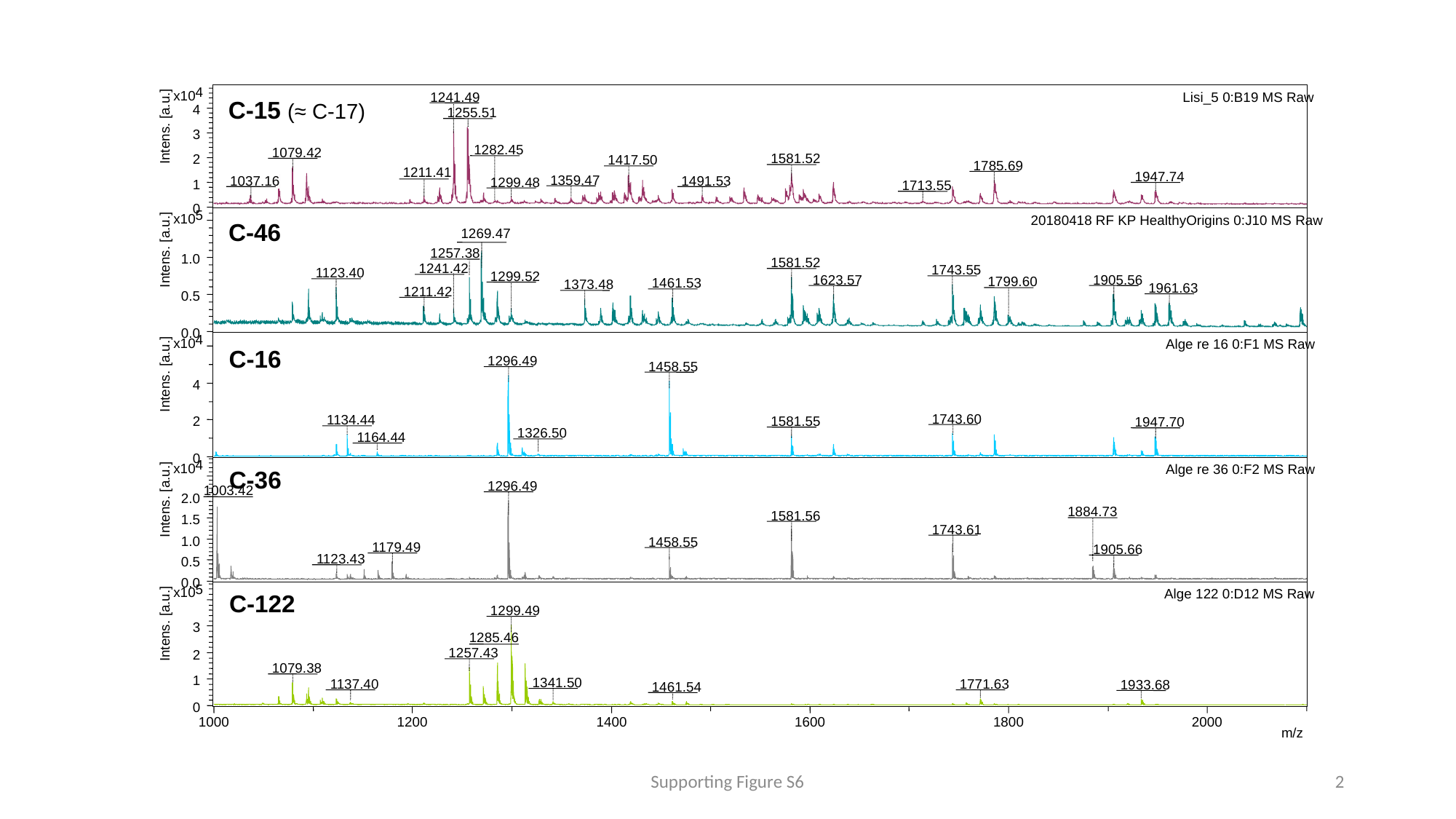

4
x10
Lisi_5 0:B19 MS Raw
1241.49
C-15 (≈ C-17)
4
1255.51
Intens. [a.u.]
3
1282.45
1079.42
1581.52
2
1417.50
1785.69
1211.41
1947.74
1359.47
1037.16
1491.53
1299.48
1
1713.55
0
5
x10
20180418 RF KP HealthyOrigins 0:J10 MS Raw
C-46
1269.47
1257.38
Intens. [a.u.]
1.0
1581.52
1241.42
1743.55
1123.40
1299.52
1623.57
1905.56
1799.60
1461.53
1373.48
1961.63
1211.42
0.5
0.0
4
x10
Alge re 16 0:F1 MS Raw
C-16
1296.49
1458.55
Intens. [a.u.]
4
1743.60
1134.44
2
1581.55
1947.70
1326.50
1164.44
0
4
x10
Intens. [a.u.]
2.0
1.5
1.0
0.5
0.0
5
x10
Intens. [a.u.]
3
2
1
0
C-36
Alge re 36 0:F2 MS Raw
1003.42
1296.49
1884.73
1581.56
1743.61
1458.55
1179.49
1905.66
1123.43
C-122
Alge 122 0:D12 MS Raw
1299.49
1285.46
1257.43
1079.38
1341.50
1137.40
1771.63
1933.68
1461.54
1000
1200
1400
1600
1800
2000
m/z
Supporting Figure S6
2

## Slide 3
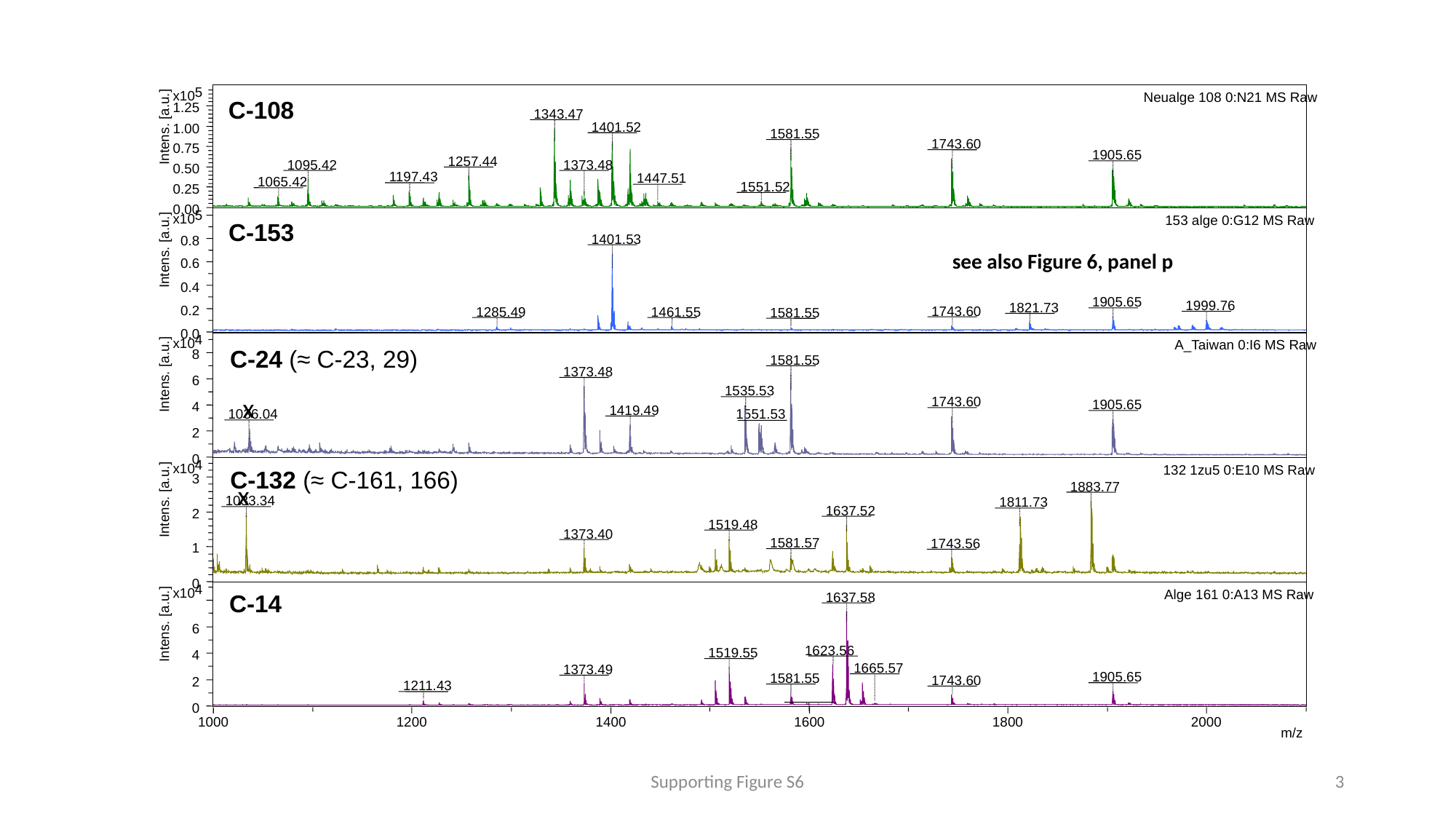

5
x10
Neualge 108 0:N21 MS Raw
1.25
1343.47
Intens. [a.u.]
1401.52
1.00
1581.55
1743.60
0.75
1905.65
1257.44
1095.42
1373.48
0.50
1197.43
1447.51
1065.42
1551.52
0.25
0.00
5
x10
153 alge 0:G12 MS Raw
1401.53
0.8
Intens. [a.u.]
0.6
0.4
1905.65
1999.76
1821.73
0.2
1743.60
1285.49
1461.55
1581.55
0.0
4
x10
A_Taiwan 0:I6 MS Raw
8
1581.55
1373.48
Intens. [a.u.]
6
1535.53
1743.60
1905.65
4
1419.49
1036.04
2
0
132 1zu5 0:E10 MS Raw
1883.77
1033.34
1811.73
1637.52
1519.48
1373.40
1581.57
1743.56
0
C-108
C-153
see also Figure 6, panel p
C-24 (≈ C-23, 29)
x
1551.53
4
x10
3
Intens. [a.u.]
2
1
4
x10
Intens. [a.u.]
6
4
2
C-132 (≈ C-161, 166)
x
C-14
Alge 161 0:A13 MS Raw
1637.58
1623.56
1519.55
1665.57
1373.49
1905.65
1581.55
1743.60
1211.43
0
1000
1200
1400
1600
1800
2000
m/z
Supporting Figure S6
3

## Slide 4
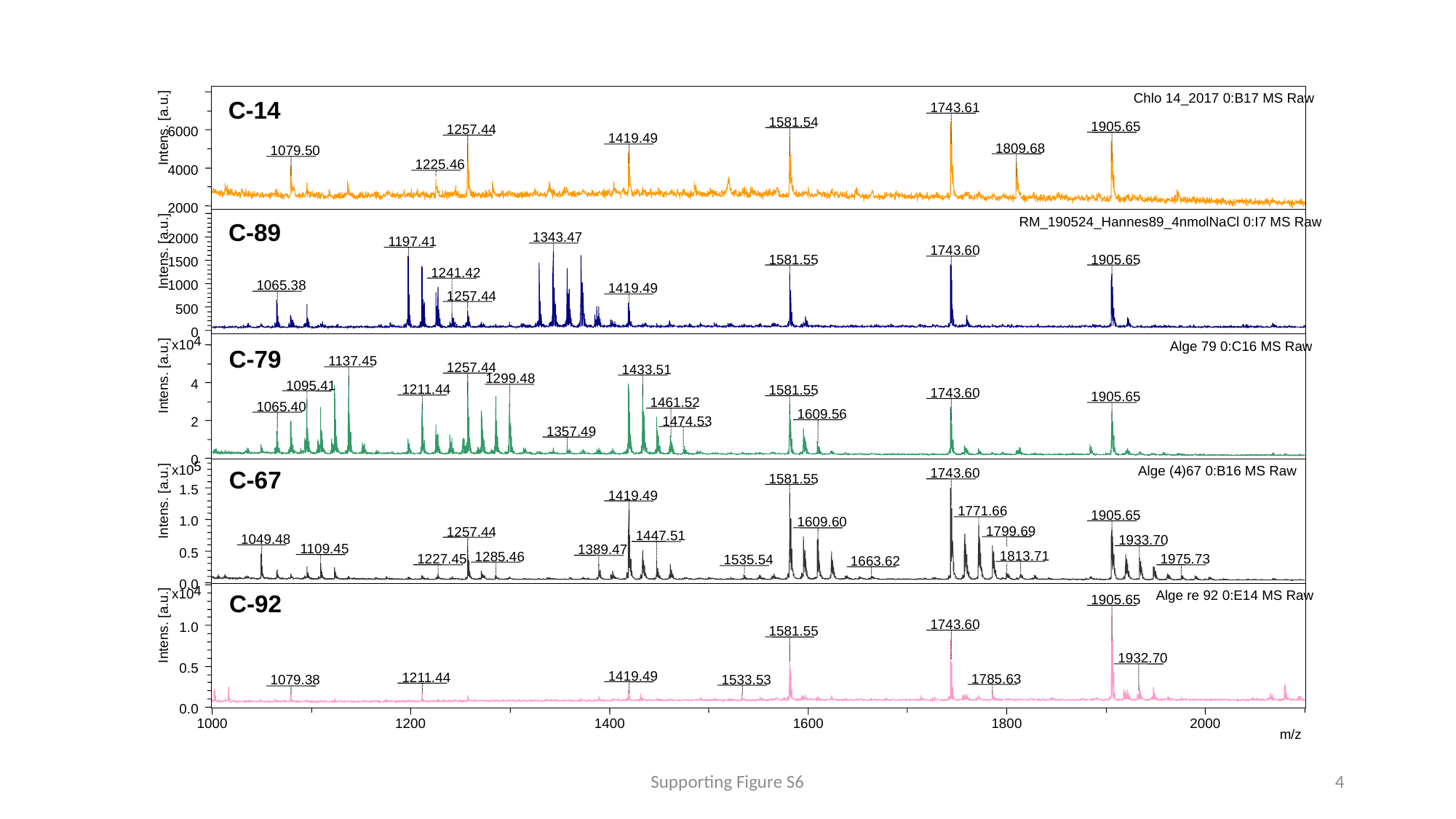

Chlo 14_2017 0:B17 MS Raw
1743.61
1581.54
1905.65
Intens. [a.u.]
1257.44
6000
1419.49
1809.68
1079.50
1225.46
4000
2000
RM_190524_Hannes89_4nmolNaCl 0:I7 MS Raw
1343.47
2000
1197.41
Intens. [a.u.]
1743.60
1581.55
1905.65
1500
1241.42
1000
1065.38
1419.49
1257.44
500
0
4
x10
Alge 79 0:C16 MS Raw
1137.45
1257.44
1433.51
Intens. [a.u.]
1299.48
4
1095.41
1211.44
1581.55
1743.60
1905.65
1461.52
1065.40
1609.56
1474.53
2
1357.49
0
1743.60
1581.55
1419.49
1771.66
1905.65
1609.60
1799.69
1257.44
1447.51
1049.48
1933.70
1109.45
1389.47
C-14
C-89
C-79
5
C-67
x10
Alge (4)67 0:B16 MS Raw
1.5
Intens. [a.u.]
1.0
0.5
1813.71
1285.46
1227.45
1975.73
1535.54
1663.62
0.0
4
C-92
x10
Alge re 92 0:E14 MS Raw
1905.65
Intens. [a.u.]
1743.60
1.0
1581.55
1932.70
0.5
1419.49
1211.44
1785.63
1533.53
1079.38
0.0
1000
1200
1400
1600
1800
2000
m/z
Supporting Figure S6
4

## Slide 5
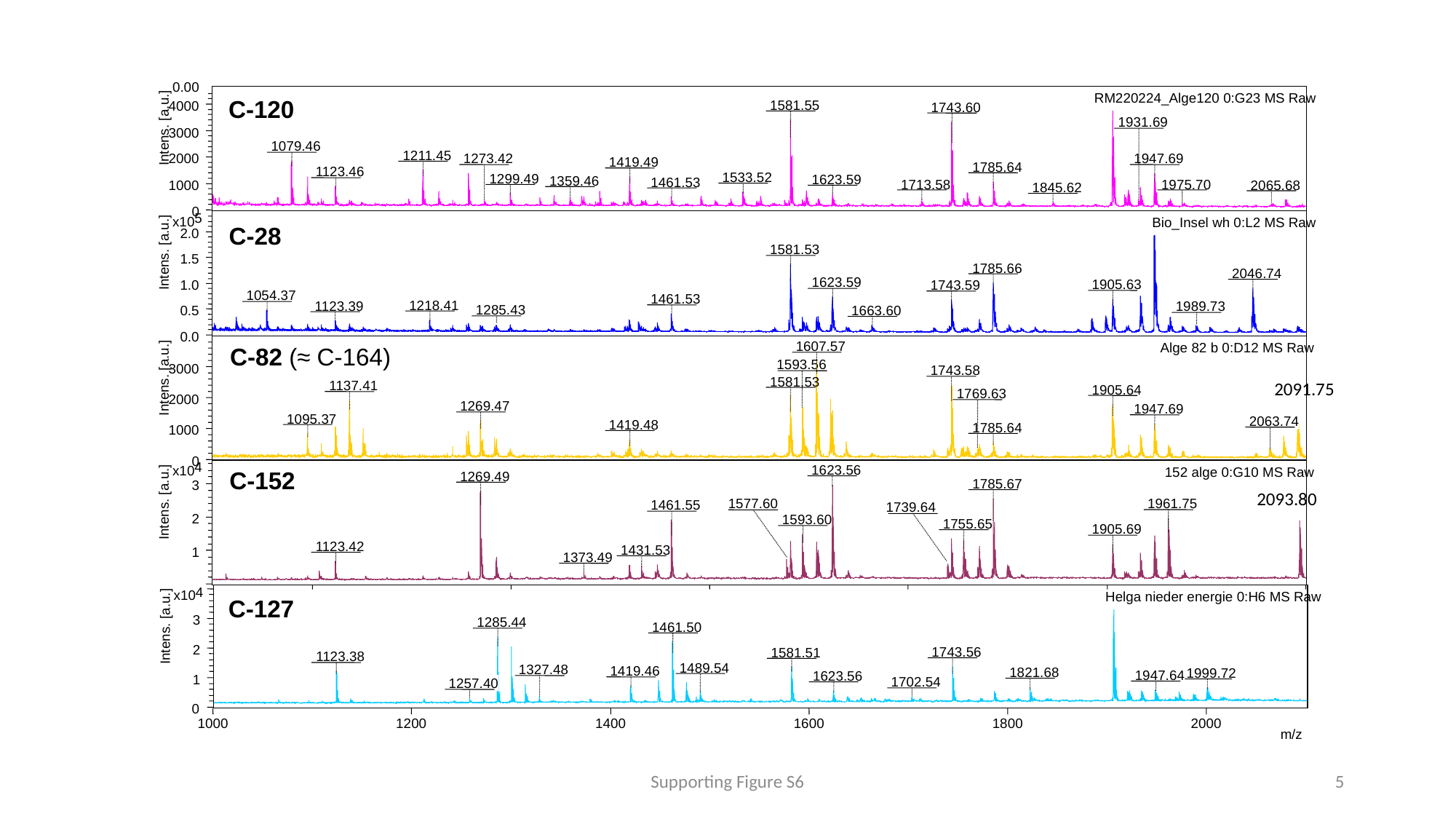

0.00
C-120
RM220224_Alge120 0:G23 MS Raw
1581.55
4000
1743.60
1931.69
Intens. [a.u.]
3000
1079.46
1211.45
2000
1273.42
1947.69
1419.49
1785.64
1123.46
1533.52
1299.49
1623.59
1359.46
1461.53
1713.58
1975.70
2065.68
1000
1845.62
0
5
x10
Bio_Insel wh 0:L2 MS Raw
C-28
2.0
1581.53
Intens. [a.u.]
1.5
1785.66
2046.74
1623.59
1905.63
1.0
1743.59
1054.37
1461.53
1218.41
1123.39
1989.73
1285.43
0.5
1663.60
0.0
C-82 (≈ C-164)
1607.57
Alge 82 b 0:D12 MS Raw
1593.56
3000
1743.58
Intens. [a.u.]
2091.75
1581.53
1137.41
1905.64
1769.63
2000
1269.47
1947.69
1095.37
2063.74
1419.48
1785.64
1000
0
4
C-152
1623.56
4
x10
Helga nieder energie 0:H6 MS Raw
C-127
3
1285.44
Intens. [a.u.]
1461.50
2
1743.56
1581.51
1123.38
1489.54
1327.48
1419.46
1821.68
1999.72
1947.64
1623.56
1
1702.54
1257.40
x10
152 alge 0:G10 MS Raw
1269.49
1785.67
3
2093.80
1577.60
Intens. [a.u.]
1739.64
1961.75
1461.55
2
1593.60
1755.65
1905.69
1123.42
1431.53
1
1373.49
0
1000
1200
1400
1600
1800
2000
m/z
Supporting Figure S6
5

## Slide 6
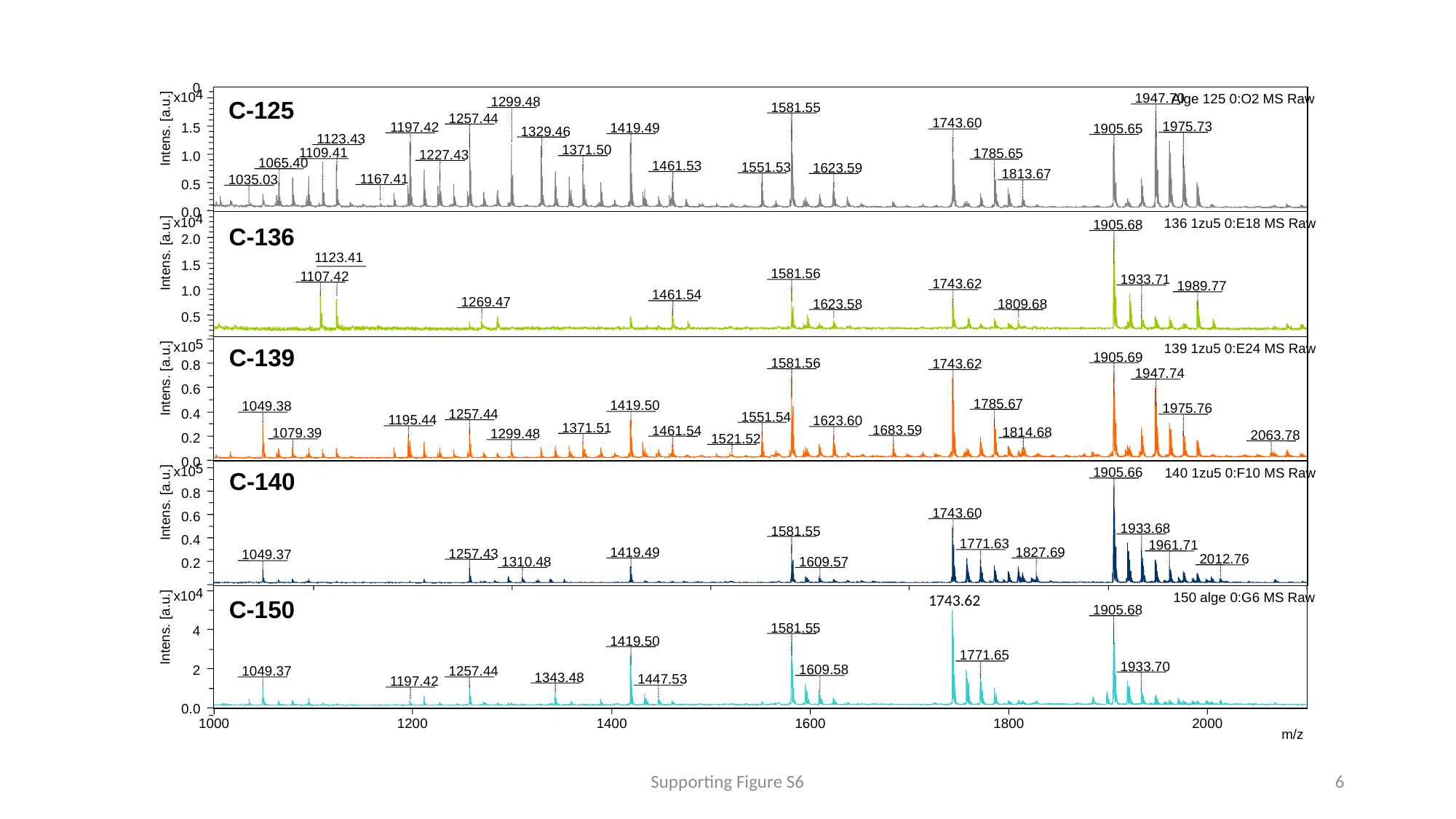

0
4
x10
C-125
1947.70
Alge 125 0:O2 MS Raw
1299.48
1581.55
1257.44
1743.60
1975.73
1197.42
Intens. [a.u.]
1.5
1419.49
1905.65
1329.46
1123.43
1109.41
1371.50
1785.65
1227.43
1.0
1065.40
1461.53
1551.53
1623.59
1813.67
1167.41
1035.03
0.5
0.0
4
x10
136 1zu5 0:E18 MS Raw
1905.68
C-136
2.0
1123.41
Intens. [a.u.]
1.5
1581.56
1107.42
1933.71
1743.62
1989.77
1.0
1461.54
1269.47
1623.58
1809.68
0.5
5
C-139
x10
139 1zu5 0:E24 MS Raw
1905.69
1581.56
1743.62
0.8
1947.74
Intens. [a.u.]
0.6
1785.67
1419.50
1049.38
1975.76
0.4
1257.44
1551.54
1195.44
1623.60
1371.51
1683.59
1461.54
1814.68
1079.39
1299.48
2063.78
0.2
1521.52
0.0
5
C-140
x10
1905.66
140 1zu5 0:F10 MS Raw
0.8
Intens. [a.u.]
1743.60
0.6
1933.68
1581.55
0.4
1771.63
1961.71
1419.49
1827.69
1257.43
1049.37
2012.76
1310.48
1609.57
0.2
4
1743.62
x10
C-150
150 alge 0:G6 MS Raw
1905.68
Intens. [a.u.]
1581.55
4
1419.50
1771.65
1933.70
1609.58
2
1049.37
1257.44
1343.48
1447.53
1197.42
0.0
1000
1200
1400
1600
1800
2000
m/z
Supporting Figure S6
6

## Slide 7
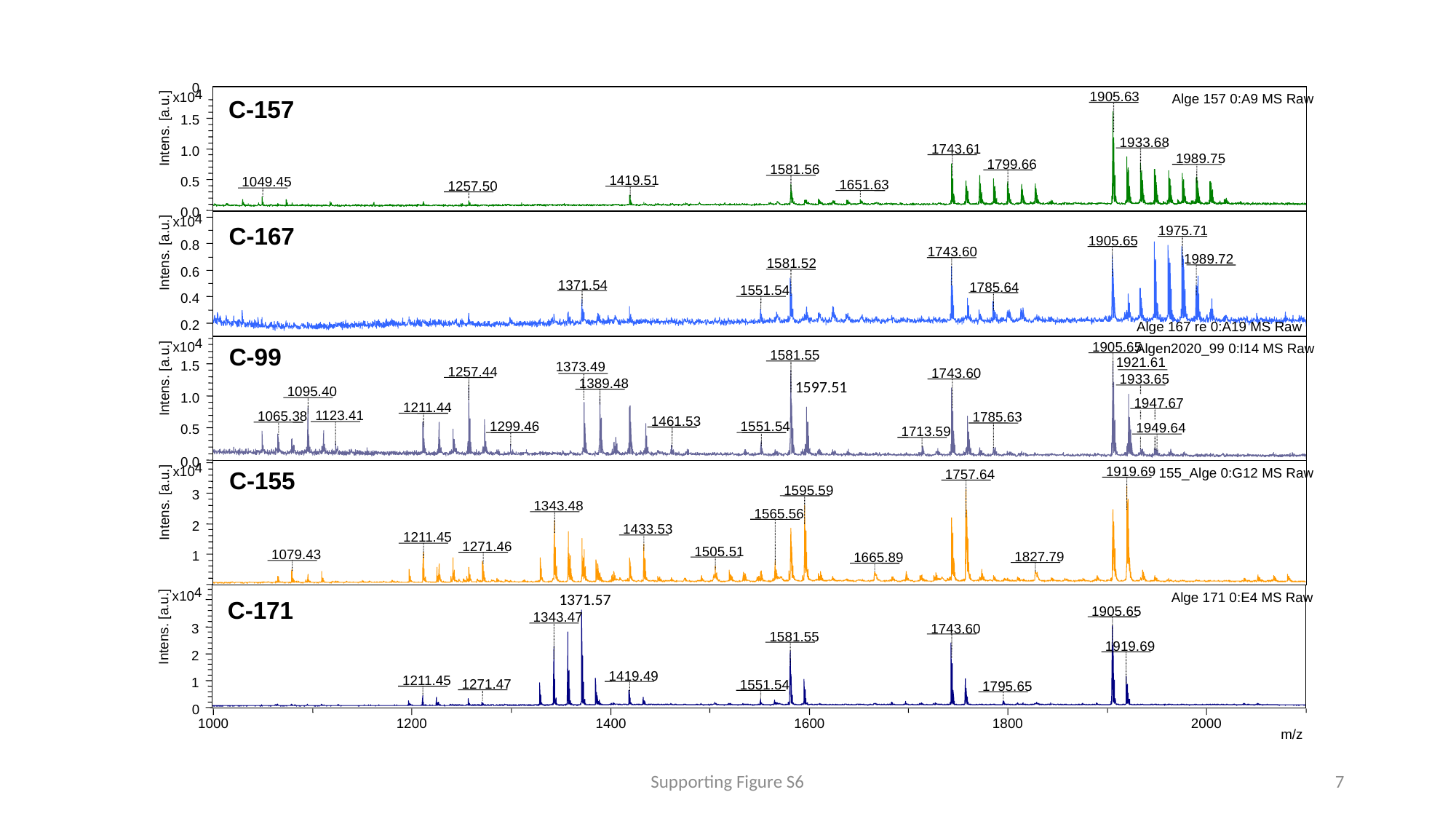

0
4
1905.63
x10
C-157
Alge 157 0:A9 MS Raw
1.5
Intens. [a.u.]
1933.68
1743.61
1.0
1989.75
1799.66
1581.56
1419.51
0.5
1049.45
1651.63
1257.50
0.0
4
x10
C-167
1975.71
1905.65
0.8
1743.60
Intens. [a.u.]
1989.72
1581.52
0.6
1371.54
1785.64
1551.54
0.4
0.2
Alge 167 re 0:A19 MS Raw
4
C-99
x10
1905.65
Algen2020_99 0:I14 MS Raw
1581.55
1921.61
1373.49
1.5
1257.44
1743.60
Intens. [a.u.]
1933.65
1597.51
1389.48
1095.40
1.0
1947.67
1211.44
1123.41
1065.38
1785.63
1461.53
1299.46
1551.54
1949.64
0.5
1713.59
0.0
4
C-155
x10
1919.69
155_Alge 0:G12 MS Raw
1757.64
1595.59
3
Intens. [a.u.]
1343.48
1565.56
2
1433.53
1211.45
1271.46
1505.51
1079.43
1
1827.79
1665.89
4
1371.57
x10
Alge 171 0:E4 MS Raw
C-171
1905.65
1343.47
Intens. [a.u.]
1743.60
3
1581.55
2
1419.49
1211.45
1
1271.47
1551.54
1795.65
1919.69
0
1000
1200
1400
1600
1800
2000
m/z
Supporting Figure S6
7

## Slide 8
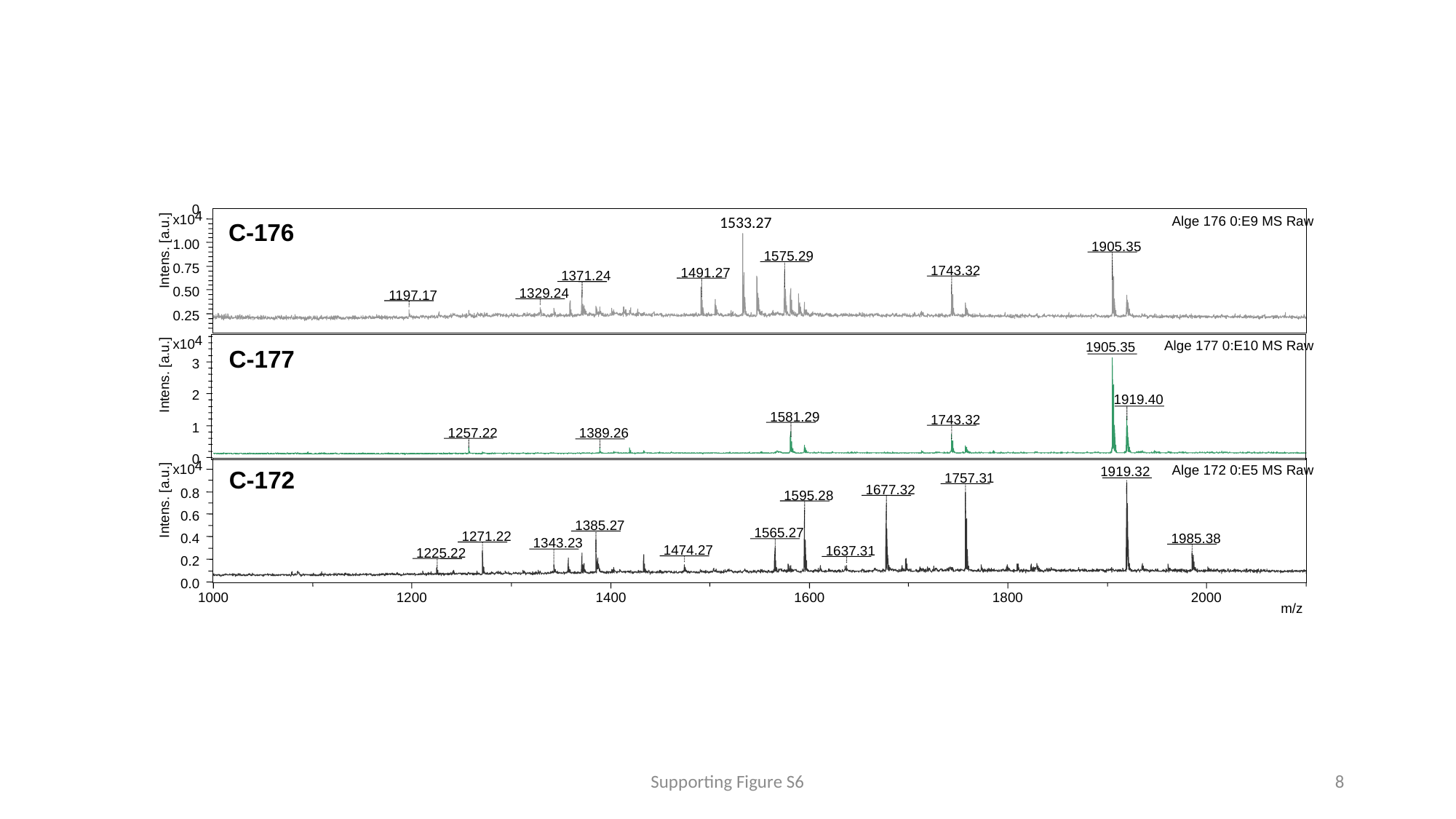

0
1533.27
4
x10
C-176
Alge 176 0:E9 MS Raw
1.00
1905.35
Intens. [a.u.]
1575.29
0.75
1743.32
1491.27
1371.24
0.50
1329.24
1197.17
0.25
4
1905.35
x10
Alge 177 0:E10 MS Raw
C-177
3
Intens. [a.u.]
1919.40
2
1581.29
1743.32
1
1257.22
1389.26
0
4
1919.32
C-172
x10
Alge 172 0:E5 MS Raw
1757.31
1677.32
0.8
1595.28
Intens. [a.u.]
0.6
1385.27
1565.27
1271.22
0.4
1985.38
1343.23
1474.27
1637.31
1225.22
0.2
0.0
1000
1200
1400
1600
1800
2000
m/z
Supporting Figure S6
8
