# Supplementary material for: The Diversity of N-Glycans of Chlorella Food Supplements Challenges Current Species Classification
Source: Foods. 2024 Oct 7;13(19):3182. doi: 10.3390/foods13193182 (PMC11482596; doi:10.3390/foods13193182)
Supplement: Supplementary file 1 [file foods-13-03182-s001.zip › Mocsai Supporting sequences.pdf]

Supporting sequences for

The diversity of N-glycans of *Chlorella* food supplements challenges current species classification

Réka Mócsai [1], Johannes Helm [1], Karin Polacsek [1], Johannes Stadlmann [1], Friedrich Altmann [1,*]

Department of Chemistry, BOKU University, Muthgasse 18, Vienna, Austria
rekamocsai@gmail.com (R.M.); jhelm@gmx.net (J.H.); karin.polacsek@boku.ac.at (K.P.); j.stadlmann@boku.ac.at (J.S.); friedrich.altmann@boku.ac.at (F.A.)
* Correspondence: friedrich.altmann@boku.ac.at)

Part a: Sequences from Mocsai *et al.* 2019 used for identity assessment.

Nucleotide sequences of ITS1-5.8S-ITS2 rRNA gene with flanking regions of 18S and 26S rDNA.
Identity assessment using web.expasy.org/sim/ was performed without the highly homologous regions at the 5′ and 3′ end (in brackets). A highly conserved region comprising 23 % of the compared sequence – roughly representing the 5.8S rRNA - is underlined. Most of the observed divergences reside in the regions before (ITS1) and after (ITG2) this 5.8S sequence.

>Kei_C1

(ACACACCGCCCGTCGCTCCTACCGATTGGGTGTGCTGGTGAAGTGTTCGGATTGGCGACCGGGGGCGGTCTCCGCTCTCGGCCGCCGAGAAGTTCATTAAACC
CTCCCACCTAGAGGAAGGAGAAGTCGTAACAAGG)
TTTCCGTAGGTGAACCTGCGGAAGGATCATTGAATCGATCGAATCCACACCGGTAACCACACGTCGCCCCTGTGGTGCATTCGCCGACCTCCGGCGTTTCACCCT
GGCGTCGGCCCCTGGGCTGGGGCTCTCACGAGCCGCTTTCCAGGTCCGACGGGCGCCTCCCTTGGGCTCACCCCCTGGGGCTGGCGTCGGCCAAAACCCCTGTA
TCCAACCCTTTTTTAACACACCCCAAACCACAACCAACTCTGAAGCATCTTTGGTGGCCCGGCCCCGTGCCGTCCACTCCAAACCAAAGACAACTCTCAACAACG
GATATCTTGGCTCCCGTATCGATGAAGAACGCAGCGAAATGCGATACGTAGTGTGAATTGCAGAATTCCGTGAACCATCGAATCTTTGAACGCAAATTGCGCCC
GAGGCTTCGGCCGAGGGCATGTCTGCCTCAGCGTCGGTTTACACCCTCGCCCTCCCCCACCCTGTGTGGTGTGGTTGGTGCGGATCTGGCCCTCCCGGCTCCGCT
CTCCTTGAGCGTCCGGGTTGGCTGAAGTGGAGAGGCTTGAGCATGGACCCCGTTTGTAGGGCAATGGCTTGGTAGGTAGGCACCCCCTACGCAGCCTGCCGTT
GCCCGAGGGGACTTTGCTGGAGGCCCAGCAGGAATCCGGCTGTCTTTGGCAGCCGGACTACTCACTCATTCGACCT
(GAGCTCAGGCAAGA)

>Hel_C32

(ACACACCGCCCGTCGCTCCTACCGATTGGGTGTGCTGGTGAAGTGTTCGGATTGGCGACCGGGGGCGGTCTCCGCTCTCGGCCGCCGAGAAGTTCATTAAACC
CTCCCACCTAGAGGAAGGAGAAGTCGTAACAAGG)
TTTCCGTAGGTGAACCTGCGGAAGGATCATTGAATCGATCGAATCCACACCGGTAACCACACTGTCGCCCTCGGCGGTGCATTCTCTGGCTTCGGCTGGGTTTCA
CCCCGAGCGTCGGCCCCTGGGTTGGGGTTCTCACGAGCCGCTCTCCAGGTCCGGCGGGCGCCTCCCTTGGGCTCACCCCCTGGGGCTGTCGTCGGCCAAAACCC
CTGTATCCAACCTTTTTTTAACACACCCCAAACCACAACCAACTCTGAAGCATCTTTGGTGGCCCGGCCCCGTGCCGTCCACTCCAAACCAAAGACAACTCTCAA
CAACGGATATCTTGGCTCCCGTATCGATGAAGAACGCAGCGAAATGCGATACGTAGTGTGAATTGCAGAATTCCGTGAACCATCGAATCTTTGAACGCAAATTG
CGCCCGAGGCTTCGGCCGAGGGCATGTCTGCCTCAGCGTCGGTTTACACCCTCGCCCTCCCCCCCTGTGGGGGGCGGTGCGGACCTGGCCCTCCCGGCTCCGCT
CTCTCCCGAGCGTCCGGGTTGGCTGAAGCACAGAGGCTTGAGCATGGACCCCGTTTGTAGGGCAATGGCTTGGTAGGTAGGCACCCCCTACGCAGCCTGCCGT
TGCCCGAGGGGACTTTGCTGGAGGCCCAGCAGGAATCCGGCCCTTCCCGGCCGGACTACTCACTCATTCGACCT
(GAGCTCAGGCAAGA)

>Raa_C6

(ACACACCGCCCGTCGCTCCTACCGATTGGGTGTGCTGGTGAAGTGTTCGGATTGGCGACCGGGTGCGGTCTCCGCTCTCGGCCGCCGAGAAGTTCATTAAACC
CTCCCACCTAGAGGAAGGAGAAGTCGTAACAAGG)
TTTCCGTAGGTGAACCTGCGGAAGGATCATTGAATCGATCGAATCCACTCTGTGAACCAAACGTCCCCCCTTGGGTGCGGGCTTCGGTCTGCCCCAAGGCGTCG
GTTCCCTGGCTGGGGTCTTCGGACCGCAGTTAGGTCCGGCGGGCGCGCCCTCTGGCGTGTCGGCCCTCGTGGCTGCCGCCAGTTGGGTTCGCTGGAAATTGTAT
CCAACTCAACCCACCCCAAACCACAACTTATACTGAAGCAATCGGTGAGTGCACTCTGGTGCCTCGCTCTAACCAAAGACAACTCTCAACAACGGATATCTTGGC
TCCCGTATCGATGAAGAACGCAGCGAAATGCGATACGTAGTGTGAATTGCAGAATTCCGTGAACCATCGAATCTTTGAACGCAAATTGCGCCCAAGGCTTCGGC
CGAGGGCATGTCTGCCTCAGCGTCGGCTTACCCCCTCGCTCCCCCTCTCCTTTGGAGTGGGTGAACGGATCTGGTTTTCCCGGCTACGTGCTTCTGCACGCCCGG
GTTGACTGAAGTGTAGAGGCTTGAGCATGGACCCCGTTTGTAGGGCAATGGCTTGGTAGGTAGCTTAGCTACACCGCCTGCCGTGGTCCGAGGGGACTTTGCT
GGCGGCCCAGCAGGAATTCGGGTGTTGGGTTTCCCACCCCGAAAGCTTCAAACCTTCGACCT
(GAGCTCAGGCAAGA)

>Sol_C21

(ACACACCGCCCGTCGCTCCTACCGATTGGGTGTGCTGGTGAAGTGTTCGGATTGGCGACCGGGGGCGGTCTCCGCTCTCGGCCGCCGAGAAGTTCATTAAACC
CTCCCACCTAGAGGAAGGAGAAGTCGTAACAAGG)

TTTCCGTAGGTGAACCTGCGGAAGGATCATTGAATCGATCGAATCCACACCGGTAACCACACGTCGCCCTGTGCGGTGCTGCACTCAGCCGAGTGCACTCTGCG
CAGCGTCGGCCCCTGGGCTGGGGCTCTCACGAGCCGCTTTCCAGGTCCGACGGGCGCCTCCCTTGGGCTCACCCCCCGGGGCTGTCGTCGGCCAAAACCCCTGT
ATCCAACCCCTTTTTTAACACACCCCAAACCACAACCAACTCTGAAGCATCTTTGGTGGCCCGGCCCCGTGCCGTCCACTCCAAACCAAAGACAACTCTCAACAA
CGGATATCTTGGCTCCCGTATCGATGAAGAACGCAGCGAAATGCGATACGTAGTGTGAATTGCAGAATTCCGTGAACCATCGAATCTTTGAACGCAAATTGCGC
CCGAGGCTTCGGCCGAGGGCATGTCTGCCTCAGCGTCGGTTTACACCCTCGCCCTCCCCCACCCTGTGCGGTGGGGTGCAGGTGCGGACCTGGCCCTCCCGGCT
CCGCCCCGCTGTTCTTCGAGCAGCGGTGGCGCCCGGGTTGGCTGAAGCACAGAGGCTTGAGCATGGACCCCGTTTGTAGGGCAATGGCTTGGTAGGTAGGCAC
CCCCTACGCAGCCTGCCGTTGCCCGAGGGGACTTTGCTGGAGGCCCCGCAGGAATCCGGCCCGCCTTTGCGGGCGGCCGGAGCACTCACTCATTCGACCT
(GAGCTCAGGCAAGA)

>Jar_C45

(ACACACCGCCCGTCGCTCCTACCGATTGGGTGTGCTGGTGAAGTGTTCGGATTGGCGACCGGGGGCGGTCTCCGCTCTCGGCCGCCGAGAAGTTCATTAAACC
CTCCCACCTAGAGGAAGGAGAAGTCGTAACAAGG)
TTTCCGTAGGTGAACCTGCGGAAGGATCATTGAATCGATCGAATCCACACCGGTAACCAAACGTCGCCCCCCTGTGGTGCATTCGCCGGACCCCCGGCGTTTCA
CCCTGGGCGTCGGCCCCTGGGCTGGGGCTCTCACGAGCCGCTTCTCAGGTCCGACGGGCGCCTCCCTTGGGCTCACCCCCCGGGGCTGGCGTCGGCCAAAACC
CCTGTATCCAACCCTTTTTTAACACACCCCAAACCACAACTCACTCTGAAGCATCTTTGGTGGTCGTGCCTCGTGCCCTTCCACTCCAAACCAAAGACAACTCTCAA
CAACGGATATCTTGGCTCCCGTATCGATGAAGAACGCAGCGAAATGCGATACGTAGTGTGAATTGCAGAATTCCGTGAACCATCGAATCTTTGAACGCAAATTG
CGCCCGAGGCTTCGGCCGAGGGCATGTCTGCCTCAGCGTCGGTTTACACCCTCGCCCTCCCCCACCCTGCGTGGTGGGGTGCTGGTGCGGATCTGGCCCTCCCG
GCTCCCCTCTCCCTCCCAGGCGAGGCTCCGGGTTGGCTGAAGCACAGAGGCTTGAGCATGGACCCCGTTTGTAGGGCAATGGCTTGGTAGGTAGGCACCCCCTA
CGCAGCCTGCCGTTGCCCGAGGGGACTTTGCTGGAGGCCCAGCAGGAATCCGGTCGGTCCCTGTGGCCGCCGGACCACTCACTCATCGACCT

>Gov_C35

(ACACACCGCCCGTCGCTCCTACCGATTGGGTGTGCTGGTGAAGTGTTCGGATTGGCGACCCGGGGCGGTTTCCGCCCTGGGCTGCCGAGAAGTTCATTAAACC
CTCCCACCTAGAGGAAGGAGAAGTCGTAACAAGG)
TTTCCGTAGGTGAACCTGCGGAAGGATCATTGAATCGATCGAATCCACACCGGTAACCATCCTACCCCCCCTGGCCTAACACCCCAGGGGCGCCAGTCCCCTGG
CCCGGGCCCGACCCGGTGCCCAGGTCTGGCGGGGTGGGGGCCCGTCCCCCGCCTGGTAATTTGTCCAACCTTAACACACCCCAAACGTCAAAACCAAACTGAAG
CAACTGGACTGGGCGGCCCCAGCGCCCCCCATCCGCCAAACCAAAGACAACTCTCAACAACGGATATCTTGGCTCCCGTATCGATGAAGAACGCAGCGAAATGC
GATACGTAGTGTGAATTGCAGAATTCCGTGAACCATCGAATCTTTGAACGCAAATTGCGCCCGCGGCTCCGGCCAAGGGCATGCCTGCCTCAGCGTCGGCTTTC
ACCCCCTCGCCCCCAATACATTTGGGAGCGGACCTGGCACCCTCGGGGGCCCGGCCTTTTCCAAAGGCCGCCGCCCCCGGGCCTGCTGAAGTGCAGTGGCTTGA
GCATGGACCCCGTTTGCAGGGCAATGGCTTGGTAGGTAGGCCCCGGCCTGCACCCCGCCTGCCGTTGCCTGAGGGGACTTTGCTGGGAGCCTAGCAGGAATTG
GGAGCCCCAGCCCTGGCGCTGGGCCCCAACCCCCCCCATATTTCGACCT (GAGCTCAGGCAAGG)

>Asp_C59

(ACACACCGCCCGTCGCTCCTACCGATTGGGTGTGCTGGTGAAGTGTTCGGATTGGCGACCCGGGGCGGTTTCCGCCCTGGGCTGCCGAGAAGTTCATTAAACC
CTCCCACCTAGAGGAAGGAGAAGTCGTAACAAGG)
TTTCCGTAGGTGAACCTGCGGAAGGATCATTGAATCGATCGAATCCACACCGGTAACCATCCTACCCCCCCTGGCCTAACACCCCAGGGGCGCCAGTCCCCTGG
CCCGGGCCCGACCCGGTGCCCAGGTCTGGCGGGGTGGGGGCCCGTCCCCCGCCTGGTAATTTGTCCAAGCTTAACACACCCCAAACGTCAAAACCAAACTGAA
GCAACTGGACTGGGCGGCCCCAGCGCCCCCCATCCGCCAAACCAAAGACAACTCTCAACAACGGATATCTTGGCTCCCGTATCGATGAAGAACGCAGCGAAAT
GCGATACGTAGTGTGAATTGCAGAATTCCGTGAACCATCGAATCTTTGAACGCAAATTGCGCCCGCGGCTCCGGCCAAGGGCATGCCTGCCTCAGCGTCGGCTT
TCACCCCCTCGCCCCCAATACATTTGGGAGCGGACCTGGCACCCTCGGGGGCCCGGCCTTTTCCAAAGGCCGCCGCCCCCGGGCCTGCTGAAGTGCAGTGGCTT
GAGCATGGACCCCGTTTGCAGGGCAATGGCTTGCTAGGTAGGCCCCGGCCTGCACCCCGCCTGCCGTTGCCTGAGGGGACTTTGCTGGGAGCCTAGCAGGAAT
TGGGAGCCCCAGCCCTGGCGCTGGGCCCCAACCCCCCCCATATTTCGACCT   (GAGCTCAGGCAAGG)

>Jos_C24

(ACACACCGCCCGTCGCTCCTACCGATTGGGTGTGCTGGTGAAGTGTTCGGATTGGCGACCGGGGGCGGTCTCCGCTCTCGGCCGCCGAGAAGTTCATTAAACC
CTCCCACCTAGAGGAAGGAGAAGTCGTAACAAGG)
TTTCCGTAGGTGAACCTGCGGAAGGATCATTGAATCGATCGAATCCACACCGGTAACCAAACGTCGCCCCCCTGTGGTGCATTCTCCGGACATCCGGCGTTTCAC
CCTGGGCGTCGGCCCCTGGGCTGGGGCTCTCACGAGCCGCTTTCTAGGTCCGACGGGCGCCTCCCTTGGGCTCACCCCCCGGGGCTGGCGTCGGCCAAAACCCC
TGTATCCAACCCTTTTTTAACACACCCCAAACCACAACCAACTCTGAAGCATCTTTGGTGGTCCGGCCTCGTGCCGTCCACTTCAAACCAAAGACAACTCTCAACA
ACGGATATCTTGGCTCCCGTATCGATGAAGAACGCAGCGAAATGCGATACGTAGTGTGAATTGCAGAATTCCGTGAACCATCGAATCTTTGAACGCAAATTGCG
CCCGAGGCTTCGGCCGAGGGCATGTCTGCCTCAGCGTCGGTTTACACCCTCGCCCTCCCCCACCCTGTGTGGTGGGGTGTTGGTGCGGATCTGGCCCTCCCGGC
TCCGCTCTGATGAGCGTCCGGGTTGGCTGAAGTGCAGAGGCTTGAGCATGGACCCCGTTTGTAGGGCAATGGCTTGGTAGGTAGGCACCCCCTACGCAGCCTG
CCGTTGCCCGAGGGGACTTTGCTGGAGGCCCAGCAGGAATCCGGCTGTTTCGGCAGCCGGACTACTCACTCATTCGACCT   (GAGCTCAGGCAAGA)

Supporting Sequences

>Sun_C36_A_abundant_clone

(ACACACCGCCCGTCGCTCCTACCGATTGGGTGTGCTGGTGAAGTGTTCGGATTGGCATCTGGGGGCGGTCTCCGCTTCCTGACGCCGAGAAGTTCATTAAACCC
TCCCACCTAGAGGAAGGAGAAGTCGTAACAAGG)
TTTCCGTAGGTGAACCTGCGGAAGGATCATTGAATCGATCGAATCCACTCTGGTAACCAAACGTCCCCCCCTTGGTGGCAGGGCTTGCCTTGTCCCATGGGCGCC
GGTCCCCTGGCTGGGGCCTTCGGGCCGCAGTTAGGTCCGGCGGGTGTCCCTCCGATGCTGGGGCTTTTGCCCCTCTTCGGTTGGTGATGCTGGAAATTTATATTC
AACTCAACCCACCCCAAACCTCGAATTAATCTGAAGCTGTCTTGTGTCACGCCTCGGCGTAGCACTCTAACCAAAGACAACTCTCAACAACGGATATCTTGGCTCC
CGTATCGATGAAGAACGCAGCGAAATGCGATACGTAGTGTGAATTGCAGAATTCCGTGAACCATCGAATCTTTGAACGCAAATTGCGCCCAAGGCTTCGGCCAA
GGGCATGTCTGCCTCAGCGTCGGCTTACCCCCTCACCCTCCCAATCCCTGTGATTGGGCAGAGTGGATCTGGCCCTCCCGGCTCCGTTCCAACTTGTTGGCACGC
CCGGGTCGGCTGAAGTGTAGAGGCTTGAGCATGGACCCCGTTTGTAGGGCAATGGCTTGGTAGGTAGCCTCTGGTTACATCGCCTGCCGTTGTCCGAGGGGAC
TTTGCTGGCGGCCCAGCAGGAATTTGGTGCGTGCGGTTCTCCGTCGCCCAAATGCTTCACACCTTCGACCT (GAGCTCAGGCAAGA)

> Sun_C36_B_rare_clone

(ACACACCGCCCGTCGCTCCTACCGATTGGGTGTGCTGGTGAAGTGTTCGGATTGGCAGCTTAGGGTGGCAACACCTCAGGTCTGCCGAGAAGTTCATTAAACC
CTCCCACCTAGAGGAAGGAGAAGTCGTAACAAGG)
TCTCCGTAGGTGAACCTGCGGAGGGATCATTGAATTATTAAAACCACAATGTGAACCTCAACGTTCCGTGCCCTGGCTTGCCAGTGGGGCGACATGGTCAACAC
CAGGTCGTACTCACAGCTGGGTGGGCATTGTTGCCTACTCAGTGGCGCCTTGGCATGATCATACACCAGTGCTAACCACTGATAAAACCAAACTCTGAAGTTTGA
TTGCTATTCATTGGCAATCTTAACCAAAGACAACTCTCAACAACGGATATCTTGGCTCTCGCAACGATGAAGAACGCAGCGAAATGCGATACGTAGTGTGAATTG
CAGAATTCCGTGAACCATCGAATCTTTGAACGCATATTGCGCTCGAGCCTTCGGGCAAGAGCATGTCTGCCTCAGCGTCGGTTTAATCCCTCACCCCTCCCTATTA
TGGGTGCGTTGATCATGTGATCAGCCATTGGGGTGGATCTGGCTTCCCCAATCTCACTTGTTGCGATTGGGTTGGCTGAAGCACAGAGGCTTAAGCAAGGACCC
GATATGGGCTTCAACTGGATAGGTAGCAACGGCGTATGCCGACTACACGAAGTTGTTGCTTGTGGACTTTGTTAGGAGCCGAGCAGGAACATGCCTTGTGCAT
GCCTAAACTTTCGACCT (GAGCTCAGGCAAGG)

>Ori_C28

(ACACACCGCCCGTCGCTCCTACCGATTGGGTGTGCTGGTGAAGTGTTCGGATTGGCAGCCCGGGGCGGTTCCCGCTCTGGTTTGCCGAGAAGTTCATTAAACCC
TCCCACCTAGAGGAAGGAGAAGTCGTAACAAGG)

TTTCCGTAGGTGAACCTGCGGAAGGATCATTGAATCGATCGAACCCACACCGGTAACCACACAACCCCCCCTGGCGGCACGCCCCAGGGGCGCCAGTCCCCTGG
CCCGGGCCACAACCCCGGTGCCCAGGTCTGGCGGGGTGTGCCCAGCCCCCGGGCTGGGCACGCCTGGTAATTCTGTCCAACCTCAACCCATCCCAAACCCCAAA
CCAAACTGAAGCTCGACTGGAAGGGCGGCTCTCAGCAGCCCCCCGACCACAAACCAAAGACAACTCTCAACAACGGATATCTTGGCTCCCGTATCGATGAAGAA
CGCAGCGAAATGCGATACGTAGTGTGAATTGCAGAATTCCGTGAACCATCGAATCTTTGAACGCAAATTGCGCCCGAGGCTCCGGCCAAGGGCATGCCTGCCTC
AGCGTCGGCTCACACCCCTTGCCCCCCCCACCTGCTGGGGGGAGCAGACCTGGCACCCTCGGGCCAGCCTGGATTGGCTCTCCAGTCCAGCTGTGCCCGGGCCT
GCTGAAGTGCAGAGGCTTGAGCATGGACCCCGTTTGCAGGGCAATGGCTTGGTAGGCTGGCCTTCACGGCTGAGCACCGCCTGCCGTTGCCTGAGGGGACTTT
GCTGGGAGCCCAGCAGGAATTGGGGGCAGCCCTCACCGGCCCCCCAACCCTCTCACTTCGACCT (GAGCTCAGGCAAGA)

>Ori_C46_A

(ACACACCGCCCGTCGCTCCTACCGATTGGGTGTGCTGGTGAAGTGTTCGGATTGGCAACCGGGGGCGGTCTCCGCTCCGGGTTGCTGAGAAGTTCATTAAACC
CTCCCACCTAGAGGAAGGAGAAGTCGTAACAAGG)
TTTCCGTAGGTGAACCTGCGGAAGGATCATTGAATCGATCGAATCCACACCGGTAACCAACCTACCCCCCCTGGCCTCAACCCCCAGGGGCGCCAGTCCCCTGG
CCCCGGCCCCCTGCCCCGTGCAGGGCCCGGGTGCCCAGGTCTGGCGGGGTGGCCCTCGGGCTGCCTGGTAATTGTCCAACCTCAACACACCCCAAACACCTAAC
CACACTGAAGCAATCGGAGCGGCGGCCTCGGCCCCCAATCCACAAACCAAAGACAACTCTCAACAACGGATATCTTGGCTCCCGTATCGATGAAGAACGCAGC
GAAATGCGATACGTAGTGTGAATTGCAGAATTCCGTGAACCATCGAATCTTTGAACGCAACTTGCGCCCGCGGCTCCGGCCAAGGGCATGTCTGCCTCAGCGTC
GGCACACACCCCTCGCCCCCCCCACCGGGTGGGGAGTGGACCTGGCACCCCCAGGCCTCGGCCAGCCCTCACCGGCTGTCGCTGGCCTGGGTCTGCTGAAGTG
CAGAGGCTTGAGCATGGACCCCGTTTGCAGGGCAATGGCTTGGTAGGTAGGCGCCAGCCTGCACCCCGCCTGCCGTTGCCTGAGGGGACTTTGCTGGGAGCCC
AGCAGGAATTGGGGCCCGCCCCGGCGGCCCCAACCCCTTCTCACTTCGACCT (GAGCTCAGGCAAGA)

>Ori_C46_B

(ACACACCGCCCGTCGCTCCTACCGATTGGGTGTGCTGGTGAAGTGTTCGGATTGGCGACCGGGGGCGGTCTCCGCTCTCGGCCGCCGAGAAGTTCATTAAACC
CTCCCACCTAGAGGAAGGAGAAGTCGTAACAAGG)
TTTCCGTAGGTGAACCTGCGGAAGGATCATTGAATCGATCGAATCCACACCGGTAACCACACTGTCGCCCTGGGTCGGGTGCGCACCTCTGCGTGCTGCCCGGC
CCAGCGCCGGCCCCTGGGCTGGGGCTCTCACGAGCCGCTTCTCAGGTCCGGCGGGCGTCTCCCTTGGGCTCACCCCCCGGGGCTGCCGTCGGCCAAAACCCCTG
TATCCAACCCCCTTTTTTAACACACCCCAAACCACAACCAACTCTGAAGCATCTTTGGTGGCCCGGCCCCGTGCCGTCCACTCCAAACCAAAGACAACTCTCAACA
ACGGATATCTTGGCTCCCGTATCGATGAGGAACGCAGCGAAATGCGATACGTAGTGTGAATTGCAGAATTCCGTGAACCATCGAATCTTTGAACGCAAATTGCG
CCCGAGGCTTCGGCCGAGGGCATGCCTGCCTCAGCGTCGGTTTACACCCTCGCCCTCCCCCACCGCTTGGCTGGGGTGCTGGTGCGGATCTGGCCCTCCCGGCT

Supporting Sequences

CCGGCCCTGCCTTGTGCAGGGGCGCCCGGGTTGGCTGAAGCCCAGAGGCTTGAGCATGGACCCCGTTTGCAGGGCAATGGCTTGGTAGGTAGGCACCCCCTAC
GCAGCCTGCCGTTGCCCGAGGGGTCTTTGCTGGAGGCCCAGCAGGAATTCGGCCCTCACCGGCCGAACCACTCACTCATTCGACCT (GAGCTCAGGCAAGA)

>Ori_C46_C

(ACACACCGCCCGTCGCTCCTACCGATTGGGTGTGCTGGTGAAGTGTTCGGATTGGCAGCTTAGGGTGGCAACACCTCAGGTCTGCCGAGAAGTTCATTAAACC
CTCCCACCTAGAGGAAGGAGAAGTCGTAACAAGG)
TCTCCGTAGGTGAACCTGCGGAGGGATCATTGAATTATTAAAACCACAATGTGAACCTCAACGTTCCGTGCCCTGGCTTGCCAGTGGGGCGACATGGTCAACAC
CAGGTCGTACTCACAGCTGGGTGGGCATTGTTGCCTACTCAGTGGCGCCTTGGCATGATCATACACCAGTGCTAACCACTGATAAAACCAAACTCTGAAGTTTGA
TTGCTATTCATTGGCAATCTTAACCAAAGACAACTCTCAACAACGGATATCTTGGCTCCCGTATCGATGAAGAACGCAGCGAAATGCGATACGTAGTGTGAATTG
CAGAATTCCGTGAACCATCGAATCTTTGAACGCATATTGCGCTCGAGCCTTCGGGCAAGAGCATGTCTGCCTCAGCGTCGGTTTACACCCTCACCCCTCCCTTTCT
TGGGTGTGTTGATCTTTGATCAACCATTGGGGTGGATCTGGCTTCCCCAATCTGCCTTGTAGCGGATTGGGTTGGCTGAAGCACAGAGGCTTAAGCAAGGACCC
GATATGGGCTTCAACTGGATAGGTAGCAACGGCTTGTGCCGACTACACGAAGTTGTTGCCTGTGGACTTTGCTAGAGGCCAAGCAGGAACATGCTTATGCATGC
CTAAACTTTCGACCT (GAGCTCAGGCAAGG)

>SAG_211_8k_Chlorella_sorokiniana_owndata der mit Scenedes fast idented Abschnitt

(ACACACCGCCCGTCGCTCCTACCGATTGGGTGTGCTGGTGAAGTGTTCGGATTGGCGACCGGGGGCGGTCTCCGCTCTCGGCCGCCGAGAAGTTCATTAAACC
CTCCCACCTAGAGGAAGGAGAAGTCGTAACAAGG)
TTTCCGTAGGTGAACCTGCGGAAGGATCATTGAATCGATCGAATCCACACCGGTAACCACACTGTCGCCCTCGGCGGTGCATTCTCTGGCTTCGGCTGGGTTTCA
CCCCGAGCGTCGGCCCCTGGGTTGGGGTTCTCACGAGCCGCTCTCCAGGTCCGGCGGGCGCCTCCCTTGGGCTCACCCCCTGGGGCTGTCGTCGGCCAAAACCC
CTGTATCCAACCTTTTTTTAACACACCCCAAACCACAACCAACTCTGAAGCATCTTTGGTGGCCCGGCCCCGTGCCGTCCACTCCAAACCAAAGACAACTCTCAA
CAACGGATATCTTGGCTCCCGTATCGATGAAGAACGCAGCGAAATGCGATACGTAGTGTGAATTGCAGAATTCCGTGAACCATCGAATCTTTGAACGCAAATTG
CGCCCGAGGCTTCGGCCGAGGGCATGTCTGCCTCAGCGTCGGTTTACACCCTCGCCCTCCCCCCCTGTGGGGGGCGGTGCGGACCTGGCCCTCCCGGCTCCGCT
CTCTCCCGAGCGTCCGGGTTGGCTGAAGCACAGAGGCTTGAGCATGGACCCCGTTTGTAGGGCAATGGCTTGGTAGGTAGGCACCCCCTACGCAGCCTGCCGT
TGCCCGAGGGGACTTTGCTGGAGGCCCAGCAGGAATCCGGCCCTTCCCGGCCGGACTACTCACTCATTCGACCT
(GAGCTCAGGCAAGA)

Comment: 100 % identity with UTEX1665; 1 base change with UTEX1230

>SAG211_34_owndata_GenBank_ MN194596

(ACACACCGCCCGTCGCTCCTACCGATTGGGTGTGCTGGTGAAGTGTTCGGATTGGCGACCGGGTGCGGTCTCCGCTCTCGGCCGCCGAGAAGTTCATTAAACC
CTCCCACCTAGAGGAAGGAGAAGTCGTAACAAGG)
TTTCCGTAGGTGAACCTGCGGAAGGATCATTGAATCGATCGAATCCACTCTGTGAACCAAACGTCCCCCCTTGGGTGCGGGCTTCGGTCTGCCCCAAGGCGTCG
GTTCCCTGGCTGGGGTCTTCGGACCGCAGTTAGGTCCGGCGGGCGCGCCCTCTGGCGTGTCGGCCCTCGTGGCTGCCGCCAGTTGGGTTCGCTGGAAATTGTAT
CCAACTCAACCCACCCCAAACCACAACTTATACTGAAGCAATCGGTGAGTGCACTCTGGTGCCTCGCTCTAACCAAAGACAACTCTCAACAACGGATATCTTGGC
TCCCGTATCGATGAAGAACGCAGCGAAATGCGATACGTAGTGTGAATTGCAGAATTCCGTGAACCATCGAATCTTTGAACGCAAATTGCGCCCAAGGCTTCGGC
CGAGGGCATGTCTGCCTCAGCGTCGGCTTACCCCCTCGCTCCCCCTCTCCTTTGGAGTGGGTGAACGGATCTGGTTTTCCCGGCTACGTGCTTCTGCACGCCCGG
GTTGACTGAAGTGTAGAGGCTTGAGCATGGACCCCGTTTGTAGGGCAATGGCTTGGTAGGTAGCTTAGCTACACCGCCTGCCGTGGTCCGAGGGGACTTTGCT
GGCGGCCCAGCAGGAATTCGGGTGTTGGGTTTCCCACCCCGAAAGCTTCAAACCTTCGACCT
(GAGCTCAGGCAAGA)

Comment: 100 % identity of sequence with data bank entries termed:

Chlorella sp. SAG 211-34 ("*C. sorokiniana*")

Auxenochlorella pyrenoidosa

Chlorella vulgaris

Pseudochlorella pringsheimii

Chlorella pyrenoidosa

>AY591508_Chlorella_vulgaris_SAG_211-11b

TTTCCGTAGGTGAACCTGCGGAAGGATCATTGAATCTATCGAATCCACTTTGGTAACCACTCGTCCCCCTCGTCCGATGTCGCCCTCTCTTAGGAGA
GTGCGATGCGGCGAGCGTCGGTCCCCTGGCTGTGGCTCCCCCGAGCTGTTGCTCAGGTCCGGCGGGCGTCCCTTCACATGTGGGACCCCTTCTTTTT
GAGGGGACAATCCCCTTTGGAGGATCCGACGTCGGAAATTCCAACTCAACTCAACCCACCCCAAACTGAAACTTATTCTAAAGCACCTTGTGGTTGG
CAGCTCGTCTGCCGTCCACTCCAAACCAAATACAACTCTCAACAACGGATATCTTGGCTCCCGTATCGATGAAGAACGCAGCGAAATGCGATACGTA
GTGTGAATTGCAGAATTCCGTGAACCATCGAATCTTTGAACGCAACTTGCGCCTGAGGCTTCGGCCAAAGGCATGTCTGCCTCAGCGTCGGCTCACC
CCCTCGCCTCCCCATCTCATTTGATTGGGAAGGCGGATCTGACCTTCCCGGTTCCGCCGGTCACTCGTGATTGGCGCCGGGTCGGTTGAAGCTCAGA
GGTATGAGCATGGACCCCGTTCGTAGGGTAATGGCTTGGTAGGTAGGCATTCCCTACGCATCCTGCCGTTGCCCGAGGGGACTTTGCTGGAGACCTA
GCAGGAATTCGGATGCTTGGGCACCCCCCGACACCGAAACTCTTCATTTCGACCT
(GAGCTCAGGCAAGACTACCCGCTGAACTTAA)

Supporting Sequences

Part b: Sequence of sample C-126 (identical with 99.9 % identity with SAG 211-11b and UTEX 259

>C-126

```
(ACACACCGCCCGTCGCTCCTACCGATTGGGTGTGCTGGTGAAGTGTTCGGATTGGCGACCTGGGGCGGTCTCCGCTCTCGGCCGCCGAGAAGTTCA
TTAAACCCTCCCACCTAGAGGAAGGAGAAGTCGTAACAAGG)
TTTCCGTAGGTGAACCTGCGGAAGGATCATTGAATCTATCGAATCCACTTTGGTAACCACTCGTCCCCCTCGTCCGATGTCGCCCTCTCTTAGGAGA
GTGCGATGCGGCGAGCGTCGGTCCCCTGGCTGTGGCTCCCCCGAGCTGTTGCTCAGGTCCGGCGGGCGTCCCTTCACATGTGGGACCCCTTCTTTTT
GAGGGGACAATCCCCTTTGGAGGATCCGACGTCGGAAATTCCAACTCAACTCAACCCACCCCAAACTGAAACTTATTCTAAAGCACCTTGTGGTTGG
CAGCTCGTCTGCCGTCCACTCCAAACCAAATACAACTCTCAACAACGGATATCTTGGCTCCCGTATCGATGAAGAACGCAGCGAAATGCGATACGTA
GTGTGAATTGCAGAATTCCGTGAACCATCGAATCTTTGAACGCAACTTGCGCCTGAGGCTTCGGCCAAAGGCATGTCTGCCTCAGCGTCGGCTCACC
CCCTCGCCTCCCCATCTCATTTGATTGGGAAGGCGGATCTGACCTTCCCGGTTCCGCCGGTCACTCGTGATTGGCGCCGGGTCGGTTGAAGCTCAGA
GGTATGAGCATGGACCCCGTTCGTAGGGTAATGGCTTGGTAGGTAGGCATTCCCTACGCATCCTGCCGTTGCCCGAGGGGACTTTGCTGGAGACCTA
GCAGGAATTCGGATGCTTGGGCACCCCCCGACACCGAAACTCTTCATTCGACCT
(GAG)
```
